# Regulation of age-associated insulin resistance by MT1-MMP-mediated cleavage of insulin receptor

**DOI:** 10.1038/s41467-022-31563-2

**Published:** 2022-06-29

**Authors:** Xuanming Guo, Pallavi Asthana, Susma Gurung, Shuo Zhang, Sheung Kin Ken Wong, Samane Fallah, Chi Fung Willis Chow, Sijia Che, Lixiang Zhai, Zening Wang, Xin Ge, Zhixin Jiang, Jiayan Wu, Yijing Zhang, Xiaoyu Wu, Keyang Xu, Cheng Yuan Lin, Hiu Yee Kwan, Aiping Lyu, Zhongjun Zhou, Zhao-Xiang Bian, Hoi Leong Xavier Wong

**Affiliations:** 1 School of Chinese Medicine, Hong Kong Baptist University, Hong Kong SAR, China; 2 School of Biomedical Sciences, The University of Hong Kong, Hong Kong SAR, China; 3 Centre for Systems Biology Dresden, Max Planck Institute for Molecular Cell and Biology, Dresden, Germany; 4 Institute of Molecular Medicine, University of Texas Health Science Center at Houston, Houston, TX USA; 5 Respiratory Department, Jinhua Guangfu hospital, Jinhua, China; 6 Centre for Chinese Herbal Medicine Drug Development Limited, Hong Kong Baptist University, Hong Kong SAR, China

**Keywords:** Type 2 diabetes, Insulin signalling

## Abstract

Insulin sensitivity progressively declines with age. Currently, the mechanism underlying age-associated insulin resistance remains unknown. Here, we identify membrane-bound matrix metalloproteinase 14 (MT1-MMP/MMP14) as a central regulator of insulin sensitivity during ageing. Ageing promotes MMP14 activation in insulin-sensitive tissues, which cleaves Insulin Receptor to suppress insulin signaling. MT1-MMP inhibition restores Insulin Receptor expression, improving insulin sensitivity in aged mice. The cleavage of Insulin Receptor by MT1-MMP also contributes to obesity-induced insulin resistance and inhibition of MT1-MMP activities normalizes metabolic dysfunctions in diabetic mouse models. Conversely, overexpression of MT1-MMP in the liver reduces the level of Insulin Receptor, impairing hepatic insulin sensitivity in young mice. The soluble Insulin Receptor and circulating MT1-MMP are positively correlated in plasma from aged human subjects and non-human primates. Our findings provide mechanistic insights into regulation of insulin sensitivity during physiological ageing and highlight MT1-MMP as a promising target for therapeutic avenue against diabetes.

## Abstract (web summary)

Insulin sensitivity declines with age via unclear mechanisms. Here, the authors show that the activity of membrane type 1 matrix metalloproteinase (MT1-MMP) is increased with ageing, leading to cleavage of the insulin receptor, and show that metabolic effects can be rescued by MT1-MMP inhibition in mice.

## Introduction

Aging in human is associated with the development of age-associated pathologies, including insulin resistance and hyperglycemia. It is well-known that the prevalence of glucose intolerance and type 2 diabetes increases with age. However, the mechanism underlying age-associated insulin resistance is not well understood. An improved understanding of physiological insulin signaling and cellular insulin resistance may pave the way for innovative therapeutic strategies against type 2 diabetes and other age-associated pathologies related to insulin resistance.

Impaired insulin signaling is a well-known cause for insulin resistance. The dysregulation of insulin signaling is mainly attributable to reduced tyrosine kinase activity of insulin receptor (IR) and decreased surface IR presentation^1–3^. The impaired activation of IR activity has been linked to the ectopic accumulation of lipid through activation of PKCε^4^. However, the mechanisms underlying reduced IR presentation is far less explored. The ectodomain of IR has been shown to be cleaved^5–7^; importantly, the levels of its soluble fragment IR (sIR) composed of α-subunits linked with the extracellular region of β-subunits has been shown to correlate with insulin resistance in diabetic human patients and has even been suggested as a potentially superior glycaemic marker than glycoalbumin and hemoglobin A1c^7,8^. In line with the findings in diabetic patients, reduced expression of IR in the liver along with more soluble IR fragments in the plasma were detected in both mice on high-fat diet and diabetic mice (*db/db* mice)^5^. As of yet, however, the molecular mechanisms responsible for the generation of sIR remain unclear and the major contributor to IR cleavage is still unknown. Despite the close association between sIR and diabetes, it remains unclear if diabetes is the only pathological situation in which IR cleavage is increased and the extent to which IR cleavage contributes to insulin resistance. As such, identification of the major sheddases involved in this regulatory mechanism would further our mechanistic understanding of insulin resistance.

Ectodomain shedding is one of the known mechanisms for regulating the expression of cell surface receptor. Membrane type 1 matrix metalloproteinase (MT1-MMP/MMP14), a membrane-bound Zn-containing endopeptidase, is actively involved in this process. Physiologically, it cleaves a wide variety of substrates ranging from the extracellular matrix to growth factor receptors. MT1-MMP deficient mice exhibit various age-associated phenotypes and die from cachexia at around 3–4 weeks of age^9–11^. One of the characteristic phenotypes observed in *Mmp14*^−*/−*^ mice is hypoglycemia^11,12^, a condition with the dramatic reduction in blood glucose. Mutations in *MMP14* have been associated with human obese and diabetic traits^13^. Importantly, a clinical trial showed that treatment with doxycycline, a broad-spectrum MMP inhibitor, improved muscle insulin sensitivity in patients with type 2 diabetes^14^. These results reveal the potential function of MT1-MMP in the regulation of insulin function and glucose metabolism; however, the exact role of MT1-MMP in insulin resistance and the molecular mechanism by which MT1-MMP regulates insulin sensitivity are largely unknown.

Herein, we show that MT1-MMP directly cleaves the ectodomain of IR, releasing sIR and inhibiting insulin signaling. This cleavage is increased in both physiological aging and diabetic conditions. Inhibition of MT1-MMP restores IR expression, improving insulin sensitivity and glucose tolerance in both aged mice and diabetic mouse models. Our results reveal MT1-MMP as a major sheddase of IR and highlight MT1-MMP as a promising therapeutic target for the management of diabetes and other age-associated pathologies related to insulin resistance.

## Results

### Loss of MT1-MMP prevents aging- and obese-associated insulin resistance

To understand the regulatory role of MT1-MMP in insulin sensitivity, we assessed the metabolic homeostasis of mice deficient in MT1-MMP. As MT1-MMP deficient mice exhibited premature death at around 3–4 weeks after birth, the function of MT1-MMP in insulin resistance was examined in *Mmp14* haploinsufficient mice that did not display any gross postnatal phenotypes^9–11^. Consistent with previous studies, young *Mmp14*^+/−^ mice were phenotypically indistinguishable from wild-type mice^9–11^. Compared to the WT littermates, *Mmp14*^+/−^ mice fed a chow diet for 6 months exhibited comparable body weight and similar levels of plasma glucose (Supplementary Fig. 1a, b)^9,10^. In alignment with previous studies^13,15^, *Mmp14*^+/−^ mice exhibited a reduction in body weight and fatty adipose tissue along with reduced loss of lean tissue when they were challenged with a high-fat diet (Supplementary Fig. 2a, b), suggesting that modulation of MT1-MMP activity could reduce the incidence of obesity, a key risk factor in type 2 diabetes. *Mmp14*^+/−^ mice fed a high-fat diet had reduced levels of fasting plasma glucose and insulin (Supplementary Fig. 2c, d). Moreover, *Mmp14*^+/−^ mice on a standard chow diet displayed slightly improved glucose tolerance relative to age-matched wild-type mice (Supplementary Fig. 1c); this effect was further exacerbated in mice on high-fat diet (Supplementary Fig. 2e). Coupled with improved glucose tolerance, *Mmp14*^+/−^ mice on high-fat diet exhibited lower blood insulin levels compared with wild-type littermate controls in the glucose-tolerance tests (Supplementary Fig. 2f). Furthermore, the reduction in blood glucose induced by injection of insulin was also greater in *Mmp14*^+/−^ mice, indicative of increased systemic insulin sensitivity resulting from the hemizygous loss of MT1-MMP (Supplementary Fig. 2g). To investigate the tissue-specific effects of MT1-MMP deficiency on insulin sensitivity, we performed hyperinsulinemic/euglycemic clamp studies in WT and *Mmp14*^+/−^ mice on high-fat diet. The glucose infusion rates (GIR) and the glucose disposal rates (GDR) were considerably higher in high-fat diet *Mmp14*^+/−^ mice than in wild-type mice (Supplementary Fig. 3a-c). To further investigate the insulin-sensitizing effect conferred by MT1-MMP haploinsufficiency on skeletal muscle metabolism, the insulin-induced glucose disposal rate (IS-GDR), an indicator of insulin sensitivity of skeletal muscle and other peripheral tissues, was also measured; once again it exhibited a significant increase in *Mmp14*^+/−^ mice in relative to WT controls (Supplementary Fig. 3d). In addition, insulin-induced suppression of hepatic glucose production (HGP) reflecting hepatic insulin sensitivity, coupled with the peripheral glucose uptake in skeletal muscle and white adipose tissue, were significantly enhanced in *Mmp14*^+/−^ mice (Supplementary Fig. 3e–g). These results suggest that hemizygous depletion of *Mmp14* protects mice from glucose intolerance and enhances systemic insulin sensitivity under the condition of overnutrition-induced obesity.

**Fig. 1:**
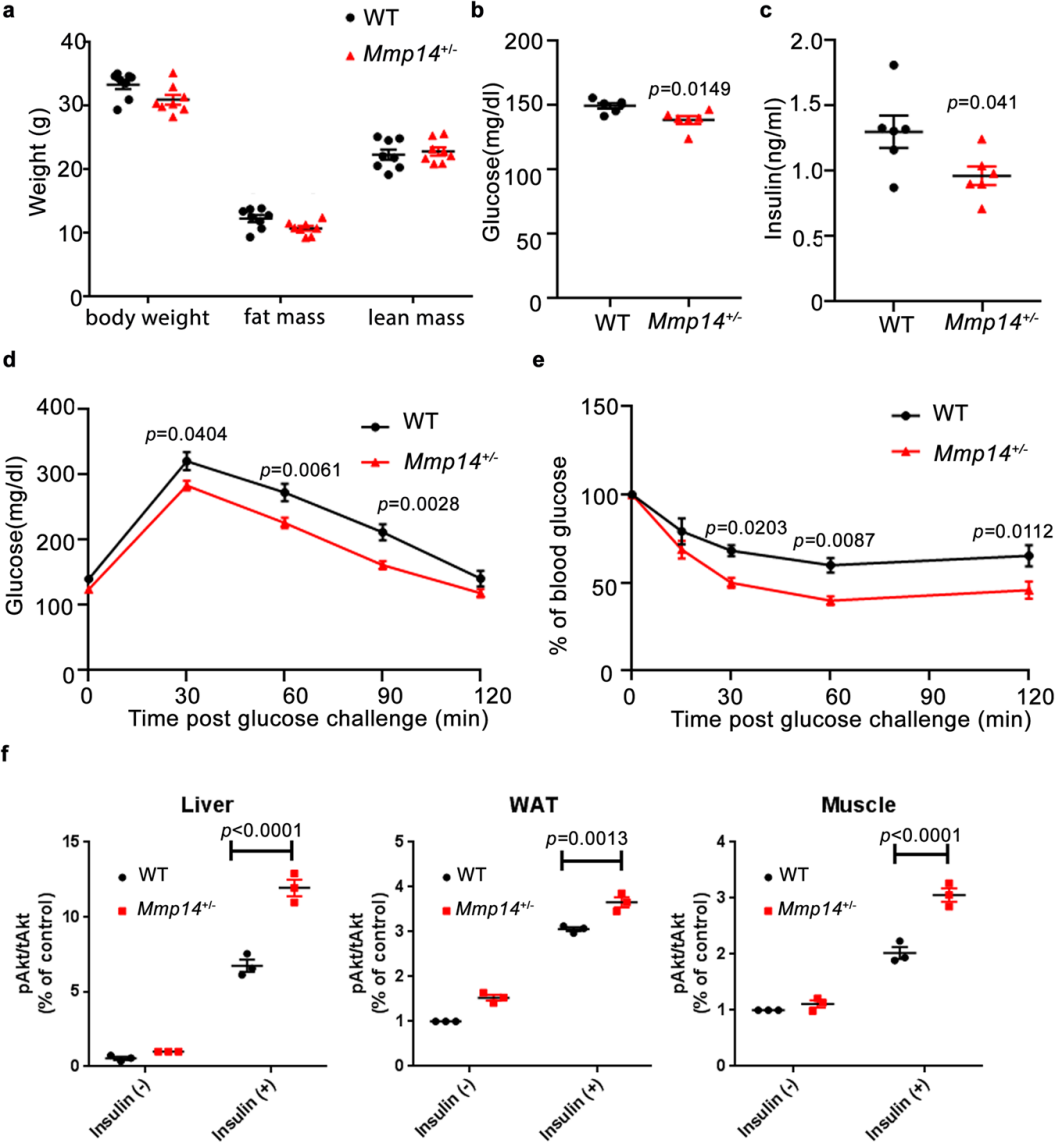
Hemizygous depletion of MT1-MMP promotes insulin sensitivity in aged mice. In all, 18-months-old WT and *Mmp14*^*+/*−^ mice on a chow diet were compared: **a** body weight and composition (*n* = 6), **b** fasting plasma glucose (*n* = 6), **c** fasting plasma insulin (*n* = 6), **d** glucose-tolerance test (*n* = 6), **e** insulin-tolerance test (*n* = 6), **f** WT and *Mmp14*^*+/-*^ mice fed chow diet at the age of 18 months were challenged with insulin. The expression of phosphorylated Akt (pAkt) in relative to the level of total Akt (tAkt) in the livers, white adipose tissues (WAT) and muscles was examined by western blotting and quantified. (*n* = 3) Representative western blots were shown in Supplementary Fig. S4D. Data are reported as average ± s.e.m.; two-sided unpaired *t* test (**a**–**c**) one-way ANOVA (**f**) or two-way ANOVA (**d**, **e**). Source data are provided as a Source Data file.

Despite no significant change in body composition, food intake, and energy expenditure, *Mmp14*^+/−^ mice fed a chow diet at 18 months of age also exhibited reduced levels of fasting plasma glucose and insulin (Fig. 1a–c and Supplementary Fig. 4a, b). Glucose tolerance and insulin-tolerance tests showed improved glucose tolerance and insulin sensitivity in *Mmp14*^+/−^ mice (Fig. 1d, e). Consistently, increased insulin signaling in key insulin-sensitive tissues, as indicated by elevated phosphorylation of Akt after the mice were challenged with insulin, was observed in aged *Mmp14*^+/−^ mice (Fig. 1f and Supplementary Fig. 4c), showing that MT1-MMP represses insulin sensitivity during physiological aging.

### Increased activation of MT1-MMP in aged and obese mice

To investigate the physiological relevance of MT1-MMP-dependent regulation of insulin sensitivity, the change of MT1-MMP expression was examined in both aged and obese mice. The protein and mRNA levels of Mmp14 were significantly upregulated in the liver of a 4-month-old-mice on a high-fat diet (Supplementary Fig. 5a, b). Interestingly, the expression of Mmp14 was elevated significantly in the liver of aged mice on chow diet, similar to the level observed in young mice on high-fat diet (Supplementary Fig. 5a, b). The upregulation of Mmp14 was similarly detected in white adipose tissue (WAT) from aged mice on chow diet (Supplementary Fig. 5c, d). We also measured MMP14 expression in WAT biopsies from lean and obese human adolescents. Consistent with results observed in mice, the expression of MMP14 increased dramatically in obese human subjects (Supplementary Fig. 6a, b). Moreover, gel zymography showed a remarkable increase in MMP activity in the liver from both aged and obese mice (Supplementary Fig. 5e, f). We further confirmed this finding by using Mmp14-specific activity assay that showed significantly increased Mmp14 activity in the liver of aged and obese mice (Supplementary Fig. 5g, h).

### MT1-MMP overexpression in mouse liver induces insulin resistance

**Fig. 2:**
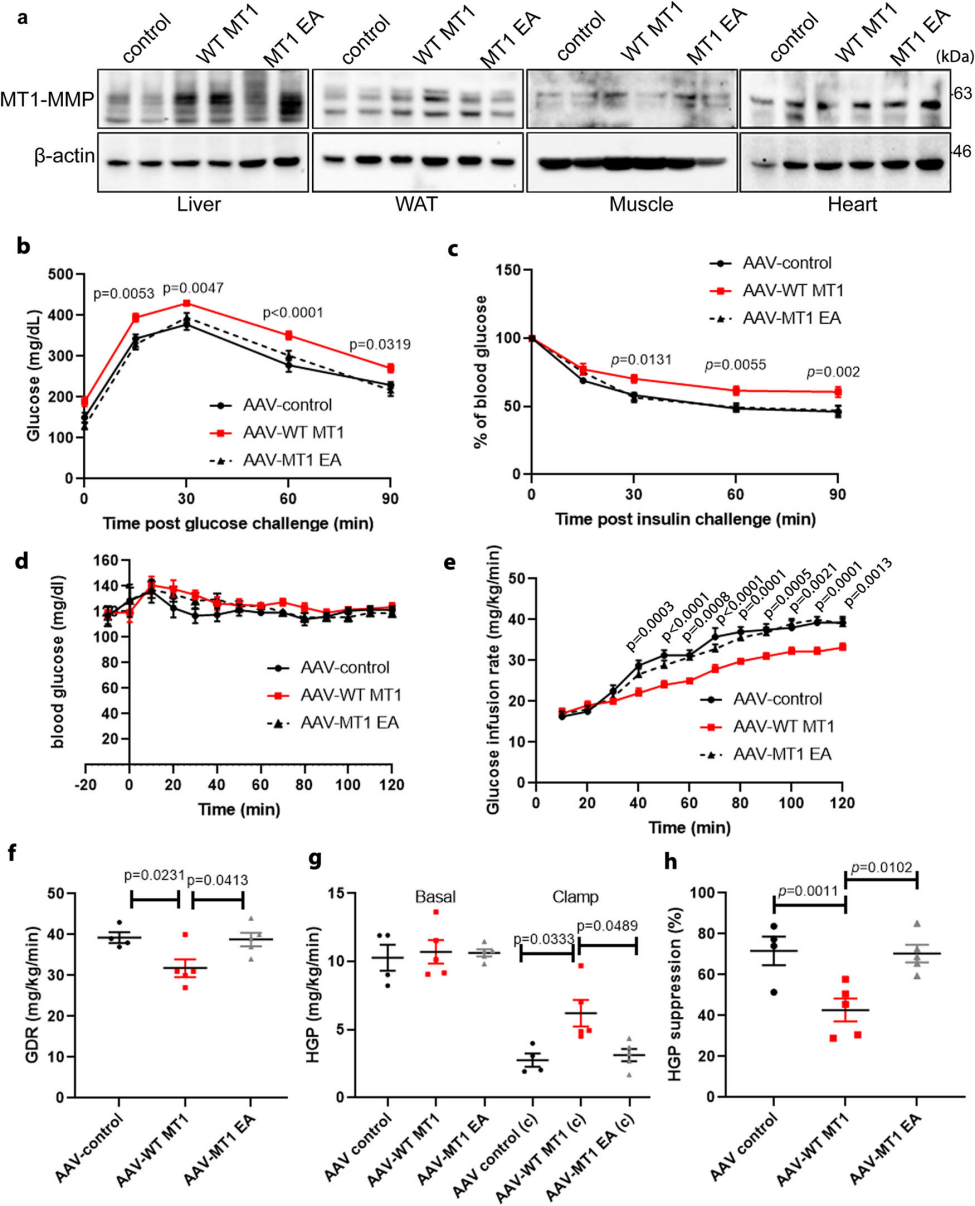
MT1-MMP ectopic expression in the liver induces insulin resistance. In all, 12-week-old male mice were injected intravenously with AAV and fed on a regular diet. **a** Western blotting analyses on the expression of MT1-MMP in the livers and other peripheral tissues including white adipose tissue (WAT), skeletal muscle and heart from mice receiving either AAV control, AAV-WT MT1, or AAV-MT1 EA (*n* = 4). **b** Plasma glucose level during the intraperitoneal glucose-tolerance tests (*n* = 6 for AAV control; *n* = 7 for AAV-WT MT1 & AAV-MT1 EA). **c** Plasma glucose level during the insulin-tolerance tests. (*n* = 6 for AAV control; *n* = 7 for AAV-WT MT1 & AAV-MT1 EA) (**d**–**h**) hyperinsulinemic/euglycemic clamp studies: plasma glucose level (**d**) and glucose infusion rate (GIR) during clamp studies (**e**), glucose disposal rate (GDR) (**f**), hepatic glucose production (HGP) (**g**), and suppression of HGP (**h**) in mice receiving either AAV control, AAV-WT MT1, or AAV-MT1 EA. (*n* = 4 for AAV control; *n* = 5 for AAV-WT MT1 & AAV-MT1 EA) Data are reported as average ± s.e.m.; one-way ANOVA (**e**–**h**) or two-way ANOVA (**b**–**e**). Source data are provided as a Source Data file.

To further investigate whether the upregulation of MT1-MMP impairs insulin action in vivo, we used an adeno-associated viral vector to ectopically express either wild-type MT1-MMP, catalytic inactive MT1-MMP mutant (MT1 EA) or GFP control in the liver. Mice were studied 4 weeks after a single intravenous administration of adenovectors. The expression of MT1-MMP was specifically increased in the liver by approximately fourfolds in mice receiving *Mmp14* AAV while no significant change in MT1-MMP expression was observed in other major metabolic tissues (Fig. 2a). Despite no significant changes in food intake, weight gain and energy expenditure (Supplementary Fig. 7), mice with *Mmp14* AAV on chow diet were more glucose intolerant and insulin resistant than mice receiving either *Mmp14 E/A* or control AAV in glucose tolerance and insulin-tolerance tests (Fig. 2b, c). To further explore insulin sensitivity, hyperinsulinemic/euglycemic clamp studies were performed. The GIR and GDR were considerably lower in mice with *Mmp14* AAV (Fig. 2d–f). Insulin-induced suppression of HGP was similarly decreased in mice with *Mmp14* AAV (Fig. 2g, h). However, there was no significant changes in both serum insulin and peripheral glucose uptake in mice with *Mmp14* AAV during the clamp (Supplementary Fig. 8a, b). These results indicate that ectopic expression of MT1-MMP in the liver is sufficient to impair insulin action and the inhibitory effect of MT1-MMP on insulin sensitivity is dependent of the catalytic activity of MT1-MMP.

### MT1-MMP directly cleaves insulin receptor to suppress insulin signaling

**Fig. 3:**
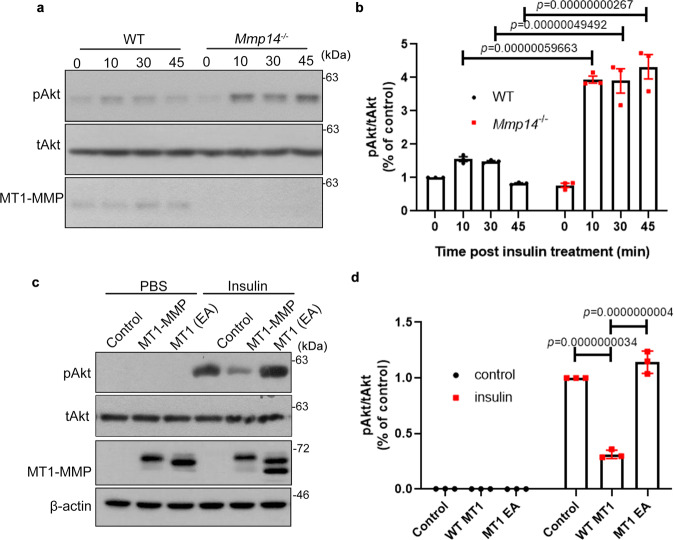
MT1-MMP suppresses insulin signaling in vitro. **a**, **b** Western blotting analyses on the expression of phosphorylated Akt (pAkt) in serum-starved wild-type and *Mmp14*^*−/*−^ primary hepatocytes upon the stimulation of insulin (**a**). Total Akt serves as a loading control. Quantification of pAkt expression in relative to tAkt expression was shown in (**b**) (*n* = 3). **c**, **d** HEK293T cells were transfected with either WT or E/A catalytic mutant MT1-MMP (MT1 E240A). The total cell lysates were subjected to western blotting analyses using antibodies indicated. Quantification of pAkt expression in relative to tAkt expression in 293T cells was shown in (**d**) (*n* = 3). Data are reported as average ± s.e.m.; one-way ANOVA (**b**, **d**). Source data are provided as a Source Data file.

To further investigate the function of MT1-MMP in insulin signaling, the level of insulin-induced Akt phosphorylation was examined in both wild-type and *Mmp14*^−/−^ primary hepatocytes. The phosphorylation of Akt in response to insulin stimulation was significantly higher in *Mmp14*^−/−^ hepatocytes (Fig. 3a, b). To further address whether MT1-MMP inactivates insulin signaling, HEK293T cells were transfected with either empty vector or WT or catalytic inactive MT1-MMP. Ectopic expression of wild-type MT1-MMP, but not catalytically inactive MT1-MMP, suppressed insulin-induced phosphorylation of Akt (Fig. 3c, d), suggesting that MT1-MMP is a negative regulator of insulin signaling.

**Fig. 4:**
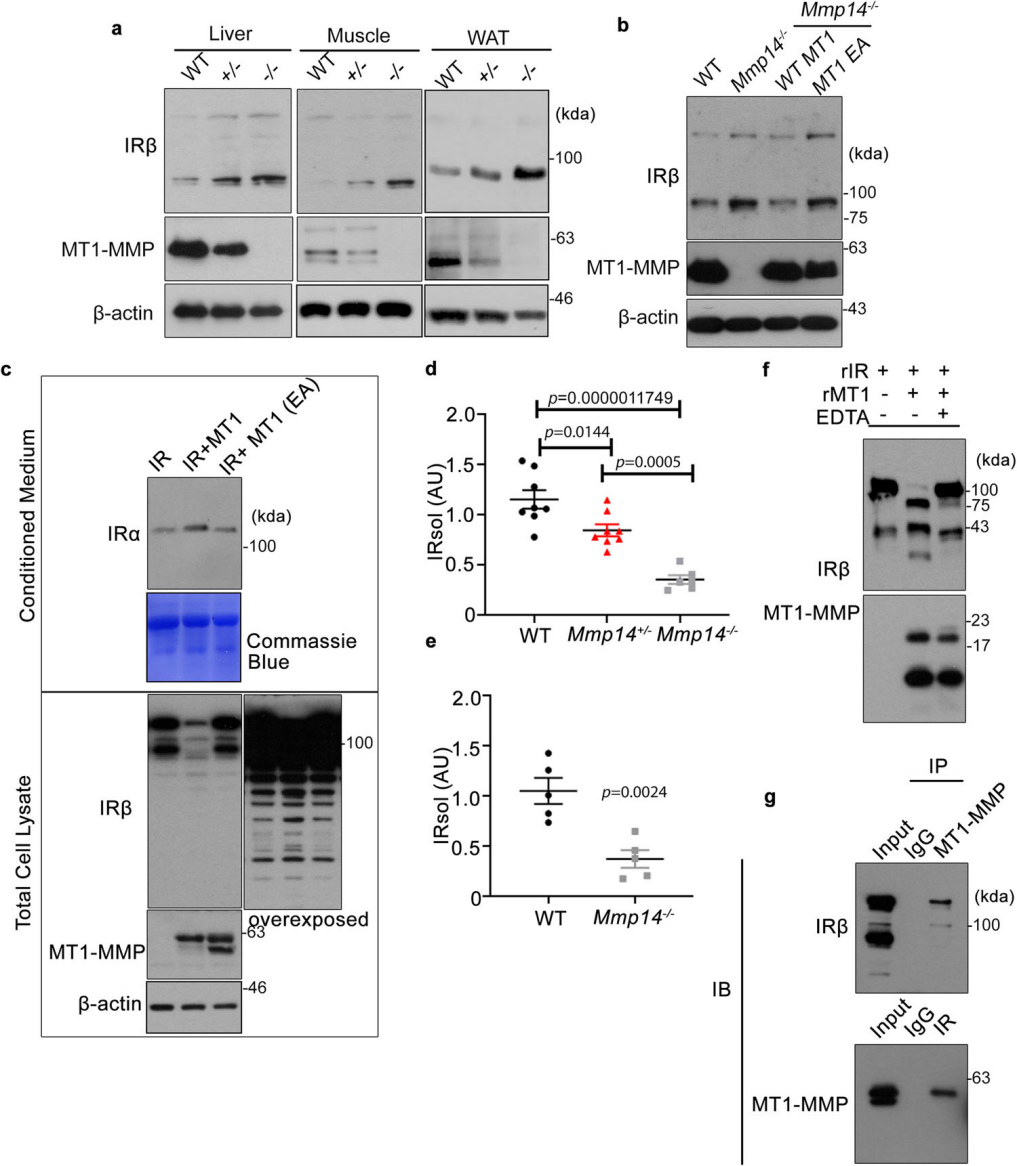
MT1-MMP directly cleaves insulin receptor to suppress insulin signaling. **a** Western blotting analyses on the expression of insulin receptor (IR) in the livers, white adipose tissues (WAT) and skeletal muscles from p15 wild-type (WT), *Mmp14*^*+/*−^, and *Mmp14*^*−/−*^ mice (*n* = 4). **b** The protein expression of IR protein in primary wild-type and *Mmp14*^*−/−*^ hepatocytes as well as *Mmp14*^*−/*−^ hepatocytes reconstituted with either wild-type MT1-MMP (WT MT1) or catalytic inactive MT1-MMP (MT1 E280A) (*n* = 3). **c** HEK293T cells were transfected with either WT or E/A catalytic mutant MT1-MMP (MT1 E280A). The expression of full-length IR and sIR was detected in total cell lysate and conditioned media respectively by western blotting (*n* = 3). **d** The level of soluble insulin receptor (sIR) was measured in the plasma from p15 wild type (WT), *Mmp14*^*+/−*^ and *Mmp14*^*−/−*^ mice. (*n* = 8 for WT & *Mmp14*^*+/−*^ mice; *n* = 6 for *Mmp14*^*−/−*^ mice); one-way ANOVA (**e**) sIR amount was measured in the conditioned media from the wild-type (WT) and *Mmp14*^*−/−*^ liver explants (*n* = 5); two-sided unpaired *t* test. **f** rIR was incubated with the recombinant catalytic domain of MT1-MMP (rMT1). The protein mixture was subjected to western blotting analyses using specific antibodies indicated (*n* = 3). **g** Co-immunoprecipitation experiments demonstrated the endogenous interaction between MT1-MMP and IR. IR and MT1-MMP immunoprecipitations (IP) were generated from liver lysates from wild-type mice and examined by western blotting analyses using indicated antibodies. IgG immunoprecipitation served as controls. Input loading showed the expression of the protein of interest in the cell lysate without the addition of an antibody (*n* = 3). Data are reported as average ± s.e.m. Source data are provided as a Source Data file.

To explore the mechanism by which MT1-MMP regulates insulin signaling, we examined the protein expression of IR. Accordingly, we obtained liver, white adipose tissue (WAT) and muscle lysates from WT as well as *Mmp14* heterozygous and knockout mice and probed their IR levels, since they are the major targets for insulin-induced glucose metabolism. Western blots against the β-chain of the insulin receptor revealed a dose-dependent elevation in the expression of IR in both liver, WAT, and muscle as *Mmp14* copy number was reduced (Fig. 4a). As the transcription of IR in these insulin-sensitive tissues was not altered by loss of MT1-MMP (Supplementary Fig. 9), these results suggest that MT1-MMP regulates IR at the protein level. Consistently, the expression of IR was also upregulated in *Mmp14*^−/−^ hepatocytes (Fig. 4b). Re-introducing wild-type MT1-MMP, but not catalytically inactive MT1-MMP (MT1 E/A), restored the expression of IR to a level similar to that observed in wild-type hepatocytes (Fig. 4b), indicating that the catalytic activity of MT1-MMP is essential for the regulation of IR expression. To test whether MT1-MMP cleaves IR, WT or activity-dead mutant MT1-MMP were transfected into HEK293T cells (Fig. 4c). Western blotting using the antibody against the β-domain of the insulin receptor revealed that the full-length IR in the total cell lysates was remarkably reduced in WT MT1-MMP transfected cells (Fig. 4c). Meanwhile, the ectodomain fragment of IR detected by the antibody against the α-chain of the insulin receptor was increased significantly in WT MT1-MMP transfected cells (Fig. 4c), suggesting that MT1-MMP sheds IR to reduce its cell surface level. To investigate whether MT1-MMP cleaves IR in vivo, we examined the level of sIR in wild-type and *Mmp14*^−/−^ mice. The amount of sIR was reduced by ~75% in the plasma from *Mmp14*^−/−^ mice (Fig. 4d). Consistently, liver explants from *Mmp14*^−/−^ mice released much less sIR than those of wild-type mice did (Fig. 4e). In contrast, overexpression of hepatic MT1-MMP did not only reduce the protein level of IR in the liver but also increased the level of sIR in the plasma (Supplementary Fig. 10a, b). Meanwhile, the transcriptional level of hepatic IR was not altered by MT1-MMP overexpression (Supplementary Fig. 10c). These results collectively confirmed the significant contribution of MT1-MMP to IR cleavage in vivo. To further confirm the direct cleavage of IR by MT1-MMP, recombinant IR was incubated with the catalytic domain of MT1-MMP (cMT1) in vitro. Full-length IR was significantly reduced and truncated fragments of IR were detected in the presence of cMT1, which was inhibited by the potent MT1-MMP inhibitor EDTA (Fig. 4f). The cleavage of IR by MT1-MMP was further substantiated by the endogenous interaction between MT1-MMP and IR in mouse primary hepatocytes (Fig. 4g). MT1-MMP was detected in IR immunoprecipitation (Fig. 4g, lower panel). Reciprocally, MT1-MMP immunoprecipitation could pull down IR (Fig. 4g, upper panel).

### Pharmacological inhibition of MT1-MMP improves aging- and obese-associated glucose homeostasis

**Fig. 5:**
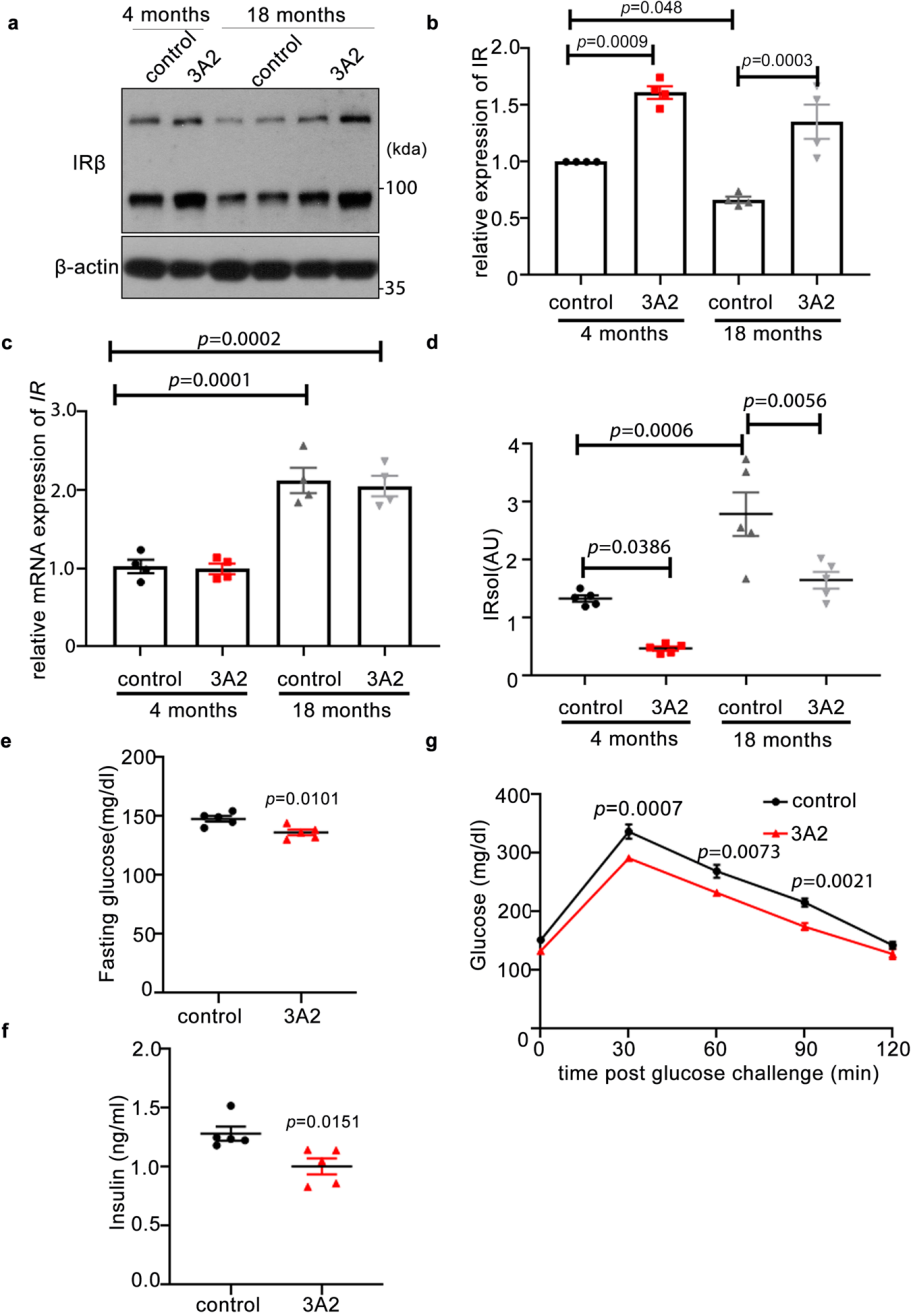
Pharmacological inhibition of MT1-MMP improves insulin sensitivity in aged mice. Male mice at 4- and 18 months old on chow diet received treatment (twice a week) with either 3A2 antibody or control IgG for 4 weeks. **a**, **b** Western blotting (**a**) and qPCR analyses (**c**) on the expression of IR in the livers. The relative expression of IR protein level in relative to β-actin levels was quantified in (**b**) (*n* = 4). **d** The level of sIR was measured in the plasma (*n* = 5). **e**–**g** Fasting plasma glucose (*n* = 5). **e** Fasting plasma insulin (*n* = 5). **f** Glucose-tolerance test (*n* = 5) (**g**) in mice. Data are reported as average ± s.e.m.; two-sided unpaired *t* test (**e**–**f**) or one-way ANOVA (**b**–**d**, **g**). Source data are provided as a Source Data file.

To further investigate the therapeutic potential of targeting MT1-MMP in the management of insulin resistance, aged mice on chow diet were administrated with 3A2, a well-characterized anti-MT1-MMP monoclonal antibody with remarkable neutralizing properties^16,17^. Interestingly, the protein expression of IR was significantly downregulated in the liver of aged mice whereas IR mRNA levels were increased (Fig. 5a–c), implying the expression of IR expression is primarily regulated in a transcription-independent mechanism. In line with reduced IR expression in the liver, the level of sIR was considerably higher in the plasma from aged mice (Fig. 5d), suggesting increased cleavage of IR in physiological aging. Specific inhibition of MT1-MMP via injection of the 3A2 monoclonal antibody restored the protein expression of both IR and sIR to a level comparable to that observed in young mice without altering mRNA expression of IR (Fig. 5a–d). Importantly, inhibition of MT1-MMP yielded significant improvements in metabolic parameters including fasting plasma glucose and insulin levels as well as glucose tolerance in aged mice (Fig. 5e, f). Similar to aged mice, inhibition of MT1-MMP activity led to normalization of hyperinsulinemia, improved glucose tolerance, enhanced insulin sensitivity and significant reduction of sIR levels in diabetic mouse models including *db/db* mice (Supplementary Fig. 11) and mice with high-fat diet-induced obesity (Supplementary Fig. 12). Taken together, our results suggest that inhibition of MT1-MMP can improve glucose homeostasis in both aged and diabetic mice.

### The correlation between soluble insulin receptor and MT1-MMP in aged primates

**Fig. 6:**
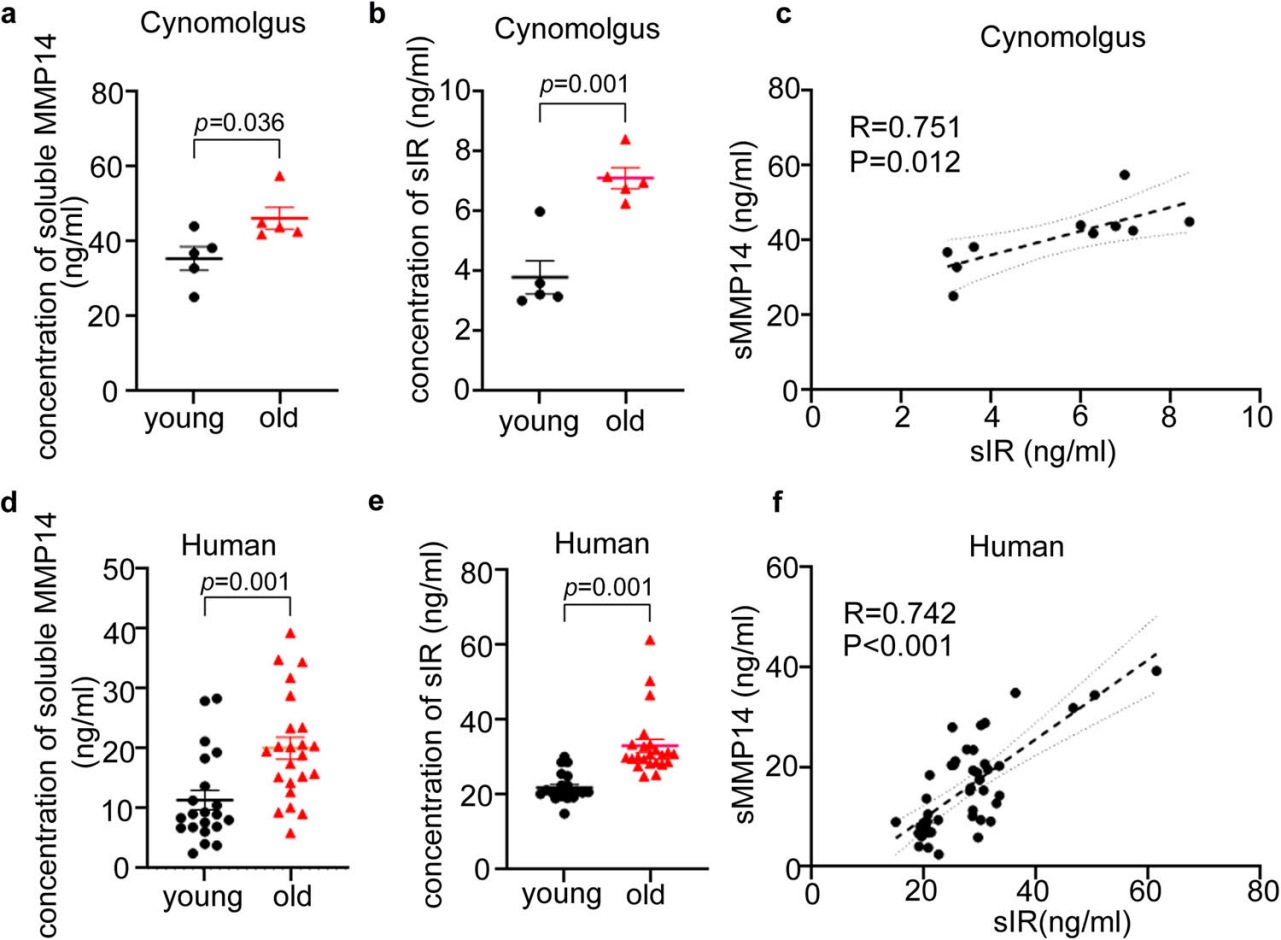
Elevated level of the soluble insulin receptor in aged primates. **a**, **b**, **d**, **e** ELISA assay reveals that there is a significant upregulation of both plasma MMP14 and soluble insulin receptor (sIR) in the plasma of healthily aged cynomolgus macaques (**a**, **b**) and human subjects (**d**, **e**) (*n* = 5 for non-human primates; 21 young and 23 elderly human subjects; two-sided *t* test). **c**, **f** There is also a positive correlation between plasma MT1-MMP and soluble insulin receptor in the plasma from aged and young non-human primates (**c**) and humans (**f**). (non-human primates: *P* = 0.012, R = 0.751, *n* = 10; human: *P* < 0.001, *R* = 0.742, *n* = 44; two-sided Spearman’s correlation). Data are reported as average ± s.e.m. Source data are provided as a Source Data file.

To determine whether our observations from murine models are physiologically relevant to primate ageing, we examined the concentrations of sIR and MMP14 in plasma derived from aged non-human primates and humans. We found that both sIR and plasma MMP14 are markedly upregulated in plasma of aged non-human primates (Fig. 6a, b) and human subjects (Fig. 6d, e). Moreover, there was a significant positive correlation between plasma MMP14 and sIR (Fig. 6c, f), reinforcing the role of MT1-MMP in insulin receptor cleavage in the primate aging process.

## Discussion

The mechanism underlying insulin resistance in physiological aging is not completely elucidated. We herein identified a previously unappreciated mechanism for the development of age-associated insulin resistance in rodent models involving ectodomain shedding of IR by MT1-MMP. We demonstrated that cleavage of IR is increased in physiological aging and this cleavage event majorly mediated by MT1-MMP contributes to the regulation of insulin sensitivity in late life.

Currently, little is known about the function of MT1-MMP in the setting of insulin resistance and diabetes. We showed that inhibition of MT1-MMP by genetic knockout or pharmacological approach improves insulin sensitivity and glucose tolerance in both aged and diabetic mice. In contrast to the improved insulin sensitivity caused by MT1-MMP depletion, ectopic MT1-MMP expression in the liver induces insulin resistance, despite no changes in body weight and composition. In line with our findings, transgenic mice with inducible MT1-MMP overexpression in the established obese adipose tissue have been reported to exhibit reduced glucose tolerance^18^. Furthermore, we found that loss of MT1-MMP enhances insulin-induced signaling in peripheral tissues in vitro and in vivo. These observations suggest that MT1-MMP exerts direct and cell-autonomous inhibitory effects on insulin sensitivity in key metabolic tissues.

We demonstrated that IR is a direct substrate of MT1-MMP, and the MT1-MMP-mediated cleavage of IR regulates insulin sensitivity and glucose tolerance. IR cleavage has been identified for years^5,7^. However, the major protease responsible for physiological IR cleavage remains to be elucidated. Deficiency in BACE1, a transmembrane aspartyl protease reported to cleave IR, only minorly reduced sIR levels and inhibition of BACE1 using a specific inhibitor does not alter plasma sIR amount^5^. In fact, we showed that inhibition of MT1-MMP leads to a remarkable reduction in sIR generation with a concomitant increase in IR expression in insulin-sensitive tissues, accompanied by robust improvement of insulin sensitivity. Interestingly, we also found that treatment of metformin, a first-line anti-diabetic medication that has been found to suppress IR cleavage in vitro^7^, significantly reduces the expression of MT1-MMP in the liver of mice with high-fat diet-induced obesity (Supplementary Fig. 13). These findings uncover MT1-MMP as a primary sheddase of IR and the cleavage of IR by MT1-MMP is physiologically relevant.

The level of sIR has been shown to correlate with insulin resistance in diabetic human patients^8^. Despite the close association between sIR and diabetes, it remains unknown whether diabetes is the only pathological situation in which IR cleavage is increased. We herein demonstrated that aging and obesity increased the expression of active MT1-MMP which in turn cleaves IR to reduce its cell surface presentation and thereby suppresses insulin signaling. The released sIR may also function as a decoy receptor to sequester the circulating insulin and decrease receptor signaling^8^. The robust improvement of insulin sensitivity resulted from the prevention of IR cleavage by inhibition of MT1-MMP nicely illustrates that MT1-MMP-mediated cleavage of IR is the major driving force in eliciting insulin resistance in both physiological aging and diabetes. Moreover, there is a highly significant correlation between sIR and plasma MMP14 in both non-human primates and humans and they are markedly upregulated in the elderly population, suggesting that the regulatory mechanism for ageing-related insulin resistance is likely conserved in both primates and non-primates. Although age-associated insulin resistance and obesity-associated insulin resistance are distinct forms of diabetes, our findings showed that the underlying cellular mechanisms that drive these diseases are unexpectedly the same. Targeting this common mechanism is therefore a potential strategy for developing efficient therapies for age-related diseases including diabetes and obesity.

## Methods

### Animals

Wild-type mice on C57BL/6J background were from the Laboratory Animal Services Centre of The Chinese University of Hong Kong. *Mmp14*^*+/*−^ mice were a kind gift from Prof. Zhou Zhongjun in the University of Hong Kong and genotyped as previously described^10^. All animals and their borne pups were housed in the animal house at Hong Kong Baptist University and maintained on a 12-h (h) light/dark cycle with constant ambient temperature (22 –24 °C) and humidity (~60%). They were fed with standard laboratory chow, and applied with water ad libitum. Animals of both sexes were used in the experiments unless it was specifically stated in the figure ligand. All mouse experiments were approved by the Committee on the Use of Human & Animal Subjects in Teaching & Research (HASC) at Hong Kong Baptist University and procedures were approved by the Department of Health under Hong Kong legislation.

### Human plasma collection

Young and elderly blood were collected for the isolation of EDTA plasma. Twenty-one young (mean = 29.2 years) and 23 healthy elderly (mean = 80.1 years) human subjects, mainly of Chinese ethnicity, were recruited from Guanfu Hospital in Jinhua, Zhejiang Province, China. They were healthy and non-obese with BMI < 24. Informed consent was obtained from each individual. Blood was collected intravenously in blood collection tubes and left at room temperature for 30 min Blood samples were then centrifuged at 300 × *g* for 10 min at 4 °C. EDTA plasma was separated and then dispensed, followed by storage at −80 °C prior to analysis. The study protocol was approved by the Research Ethics Board of Guanfu Hospital and the Health Commission of Guangdong Province in accordance with China legislation. The study was conducted in accordance with the Declaration of Helsinki.

### Plasma collection from non-human primates

For the non-human primate ageing model, plasma was collected from five young (mean = 3.8 years) and five old (mean = 18.4 years) cynomolgus macaques that were singly kept in stainless steel cages and maintained on a 12-h (h) light/dark cycle with ambient temperature (16–26 °C) and humidity (~60%). The standard maintenance diet for non-human primates (Jiangsu Synergy Pharmaceutical and Biological Engineering Co.), apples and water were available ad libitum. They were female and healthy. After 14 h of fasting, animals are anesthetized with 10 mg/kg ketamine and weighed after complete sedation. In all, 4 ml of blood was drawn from the femoral vein of the left or right leg using a 20-gauge syringe and transferred to an EDTA anticoagulation tube for each animal. The blood was immediately centrifuged at 300 × *g* for 15 min, and the plasma was stored at −20 °C. All housing conditions and procedures were approved by and in compliance with the ethical guideline of the Institutional Animal Care and Use Committee (IACUC) of Guangzhou Huazhen Biosciences Co., Ltd in Guang Zhou, Gunag dong, China. The housing facilities were accredited by the Association for Assessment and Accreditation of Laboratory Animal Care (AAALAC).

The levels of plasma insulin receptor and plasma MMP14 were measured using ELISA kits from BioVenor and Cloud-clone Corporates respectively.

### High-fat diet feeding

Wild-type mice on C57BL/6J background were fed with either a high-fat diet (45% fat; research diets D12492) or control diet (10% fat) from 6 weeks of age for 8 weeks.

### Metabolic measurement

The metabolic measurement of mice was performed as previously described^15^. Body weight and food intake were measured daily. Food intake was recorded by measuring daily changes in the amount of food content in food hoppers. Tail blood samples were collected after 4 h of food restriction and used for the measurement of plasma insulin. For the glucose-tolerance tests, food was removed for 6 h before mice were intraperitoneally injected with 1 mg/kg glucose. Tail vein blood was drawn to measure plasma insulin with a mouse insulin ELISA kit (Novus) and plasma glucose using a One Touch Ultra glucometer (LifeScan). For the insulin-tolerance tests, mice were intraperitoneally administrated with 0.5 units/kg of insulin after 6 h of fasting. Blood was drawn from tail veins for the measurement of blood glucose.

Hyperinsulinemic/euglycemic clamp was performed as in the previous study with minor modification^19^. Briefly, mice were catheterized with dual catheters (MRE-025, Braintree Scientific) for 4–5 days before the experiment. Mice fasted for 6hrs were given a constant infusion of [3-^3^H] glucose (5 μCi bolus), followed by infusion with [3-^3^H] glucose (0.05 μCi/min). After 90 min of basal sampling, the clamp began at *t* = 0 with a continuous infusion of human insulin (2.5 mU/kg/min for mice fed chow diet & 4 mU/kg/min for mice fed high-fat diet) after a bolus and the infusion of [3-^3^H] glucose was increased to 0.1 μCi/min for the rest of the experiment. Blood samples were drawn from the tail vein at every 10-min for maintaining euglycemia (100–140 mg/dl). A 10-μCi bolus of (2-deoxy-D-[1,^14^C] glucose) was given at *t* = 75 min. Blood samples were taken at 10 min intervals from *t* = 90–120 min to determine the levels of [3-^3^H] glucose and 2[^14^C]DG levels in plasma for the measurement of hepatic glucose production and peripheral glucose disposal rates. At the end of the experiment (*t* = 120 min), mice were sacrificed for the elevation of glucose uptake in different tissues. Glucose uptake was quantified by measuring the radioactivity via scintillation. The level of circulating insulin receptors in mice was measured by ELISA as previously described with slight modification^5^. Briefly, the ELISA plate (Perkin Elmer) was coated with the monoclonal antibody against the α-subunit of the insulin receptor (Invitrogen, AHR0231). Plasma samples or conditioned culture media were then incubated in antibody-coated wells at 4 °C overnight. The biotinylated version of the monoclonal antibody against IRα (Invitrogen, MA5-13764) was incubated for 2 h at room temperate. The signal amplification and detection were performed as described by the manufacturer of the ELISA plate.

### Adeno-associated virus (AAV) treatment

AAV-WT MT1-MMP and AAV-MT1 EA virus produced by the pAAV-TBG-sfGFP-WPRE vector plasmid were purchased from Obio Technology Ltd. This vector plasmid was an AAV vector of serotype 8 under the control of the thyroxine-binding globulin (TGB) promoter, a well-documented AAV vector for specific transgene expression in hepatocytes. They were delivered by tail vein injection (5.0 × 10^10^ copies per mouse). Mice were studied 4 weeks after AAV infection.

### Antibodies

The antibodies used in this study include the following: anti-MT1-MMP antibody (ab51074, Abcam; 1:2000 for western blotting); anti-insulin Rα antibody (sc-57344, Santa Cruz, 1:2000 for western blotting); anti-insulin Rβ antibody (sc-711, Santa Cruz, 1:2000 for western blotting); anti-insulin Rβ antibody (clone CT-3, MAB S65, millipore, 1:2000 for western blotting); anti-Akt (4685, Cell Signaling, 1:3000 for western blotting): anti-pAkt (4060, Cell Signaling, 1:2000 for western blotting); anti-β-actin (12262, Cell Signaling, 1:5000 for western blotting); goat anti-rabbit antibody conjugated with HRP (sc-2030, Santa Cruz, 1:2000); Rabbit anti-mouse antibody conjugated with HRP (sc-358914, Santa Cruz, 1:2000).

### DNA constructs

Plenti6/V5-DEST plasmids expressing mouse full-length MT1-MMP and its mutants tagged with Flag at the C terminus were a kind gift from Dr. Takeharu Sakamoto.

### Cell treatment

HEK293T cells obtained from Prof. Zhou Zhongjun at the University of Hong Kong were cultured in the Dulbecco’s modified Eagle’s medium (DMEM) (Gibco) supplemented with 10% fetal bovine serum (FBS) and penicillin/streptomycin (100 ng/ml). The cells have recently been tested negative for contamination of mycoplasma. For experiments with plasmid transfection, cells at 80–90% confluence were transfected using Lipofectamine 3000 (Invitrogen).

Primary hepatocytes from mice on C57BL/6J background were isolated using the collagenous perfusion method. Briefly, the liver from mice was perfused with 7 ml of liver perfusion medium at 1 ml/min, which was followed by perfusion with 5 ml of collagenase (1 mg/min). The perfused liver was minced gently in DMEM. Cells were washed with hepatocyte wash medium and purified with Percoll solution (45% v/v) by centrifugation at 72 g for 2 min. Hepatocytes were cultured in William’s E medium containing 5% FBS and penicillin/streptomycin (100 ng/ml). For the generation of lentiviral supernatants, 293T cells cultured in DMEM supplemented with 10% FBS and 100 ng ml^−1^ penicillin/streptomycin were transfected with plenti6 plasmids and packaging vectors (Addgene) using Lipofectamine 3000 (Invitrogen). Hepatocytes were infected with lentiviral supernatant in the presence of polybrene (6 μg/ml Millipore) for 18 h, followed by drug selection with Blasticidin (3 μg/ml for 2 days. Survived cells were used for the studies.

### Western blotting

Total protein was either extracted from cells or tissues using ice-cold RIPA buffer (25 mM Tris-HCl, 150 mM NaCl, 1% NP-40, 1% sodium deoxycholate, 0.1% SDS, complete protease cocktail (Roche)). Protein samples at 10 μg were separated by 7.5–15% SDS-PAGE and electrophoretically transferred to polyvinylidene difluoride membranes (Bio-rad). After blocking with 5% non-fat milk, membranes were incubated with primary antibodies overnight at 4 °C. After rinsing, membranes were incubated with appropriate secondary antibodies conjugated with HRP at room temperature for 1 h. The positive immunoreactions were detected with x-ray film (Fuji) by chemiluminescence using an ECL kit (GE Healthcare). The relative expression of proteins was quantified using Image J software (Wayne Rasband, NIH, USA). Protein bands of western blots were quantified using Image J (version 1.8.0_172).

### Real-time quantitative polymerase chain reaction (qPCR)

Total RNA was extracted from cells or tissues using TRIzol reagent (Invitrogen) according to the manufacturer’s instructions. In total, 2 μg of the total RNA of each sample was reversely transcribed into cDNA using PrimeScript RT master mix (Takara) in a total volume of 20 μl. cDNA templates were then amplified with specific primers for target genes in the ABI ViiA 7 real-time PCR system (Applied Biosystems) using 2× SYBR Green PCR Master Mix (Applied Biosystems). Expression of the gene of interest of each sample was normalized to the endogenous control GAPDH, and presented as 2-ΔΔCt using the comparative Ct method. The results were analyzed by ViiA 7 Real-time PCR system software (QuantStudio Software v1.6.1). The sequences of primers used include: *Gapdh*: GAGCCAAAAGGGTCATC (forward); GTGGTCATGAGTCCTTC (reverse); *Mmp14:*ATGTCTCCCGCCCCTCGACC (forward); TGGGTACGCAGGTCCCCTGG (reverse).

### In vitro MT1-MMP cleavage assay

The experiments were performed as previously described^20^. The recombinant catalytic domain of MT1-MMP (BML-SE259-0010) and recombinant Insulin Receptor (11081-H08H) were purchased from Enzo and Sino Biological, respectively. The rIR consists of human IR protein (1–956 amino acids). They were incubated in the assay buffer (50 mM Tris-HCl pH 7.5, 150 mM NaCl, 5 mM CaCl_2_, and 0.025% Brij35) at 37 °C for 16 h. The protein mixture was subjected to western blotting analyses.

### Statistical analyses

Each experiment was independently performed for at least three times. Animal experiments involved at least three independent and randomly chosen mice at comparable developmental stages and none of the samples were excluded from analyses. The sample size was determined from the power of the statistical test performed and was increased in accordance with the statistical variation. The statistical differences were determined using one-way analysis of variance (ANOVA) followed by Tukey’s post hoc test, Mann–Whitney *U* test, or student’s *t* test on GraphPad Prizm 8.0 software. All values are expressed as means ± s.e.m. along with the number of individual mice/samples analyzed (*n*). All data meet the normal distribution. *P* value of < 0.05 is accepted as statistically significant. GraphPad Prism V8 for Window OS was used for statistical analyses.

### Reporting summary

Further information on research design is available in the Nature Research Reporting Summary linked to this article.

## Supplementary information

Supplementary Information

Reporting Summary

## Data Availability

All data generated or analyzed during this study are included in this published article (and its supplementary information files). Source data are provided with this paper.

## Source data

Source Data
