# Supplementary material for: Regulation of age-associated insulin resistance by MT1-MMP-mediated cleavage of insulin receptor
Source: Nat Commun. 2022 Jun 29;13:3749. doi: 10.1038/s41467-022-31563-2 (PMC9242991; doi:10.1038/s41467-022-31563-2)
Supplement: Supplementary file 1 — Supplementary Information [file 41467_2022_31563_MOESM1_ESM.pdf]

Supplementary Information for

Regulation of age-associated insulin resistance by MT1-MMP-mediated cleavage of Insulin Receptor

Xuanming Guo, Pallavi Asthana, Susma Gurung, Shuo Zhang, Sheung Kin Ken Wong, Samane Fallah, Chi Fung Willis Chow, Sijia Che, Lixiang Zhai, Zening Wang, Xin Ge, Zhixin Jiang, Jiayan Wu, Yijing Zhang, Xiaoyu Wu, Keyang Xu, Cheng Yuan Lin, Hiu Yee Kwan, Aiping Lyu, Zhongjun Zhou, Zhao-Xiang Bian, Hoi Leong Xavier Wong

* correspondence should be addressed to Wong HLX (xavierwong@hkbu.edu.hk)

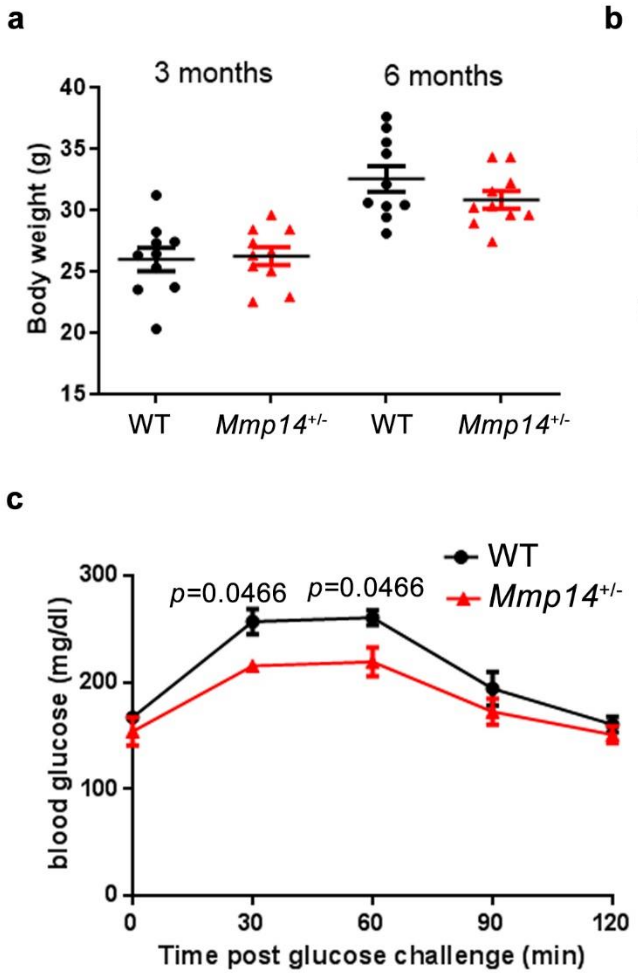

Supplementary Fig. 1. ***Mmp14*$^{+/-}$ mice are indistinguishable from wild-type mice on standard chow diet**

(a) Body weight of wild-type (WT) and *Mmp14*$^{+/-}$ mice fed with standard chow diet at 3 months and 6 months of age. (n=10) **(b)** Fasting plasma glucose of WT and *Mmp14*$^{+/-}$ mice provided with standard diet at 3 months of age. (n=10) **(c)**Plasma glucose of WT and *Mmp14*$^{+/-}$ mice on chow diet at 3 months of age in the glucose tolerance test. (n=4) Values are expressed as mean ± SEM two-way ANOVA for **(c).** Source data are provided as a Source Data file.

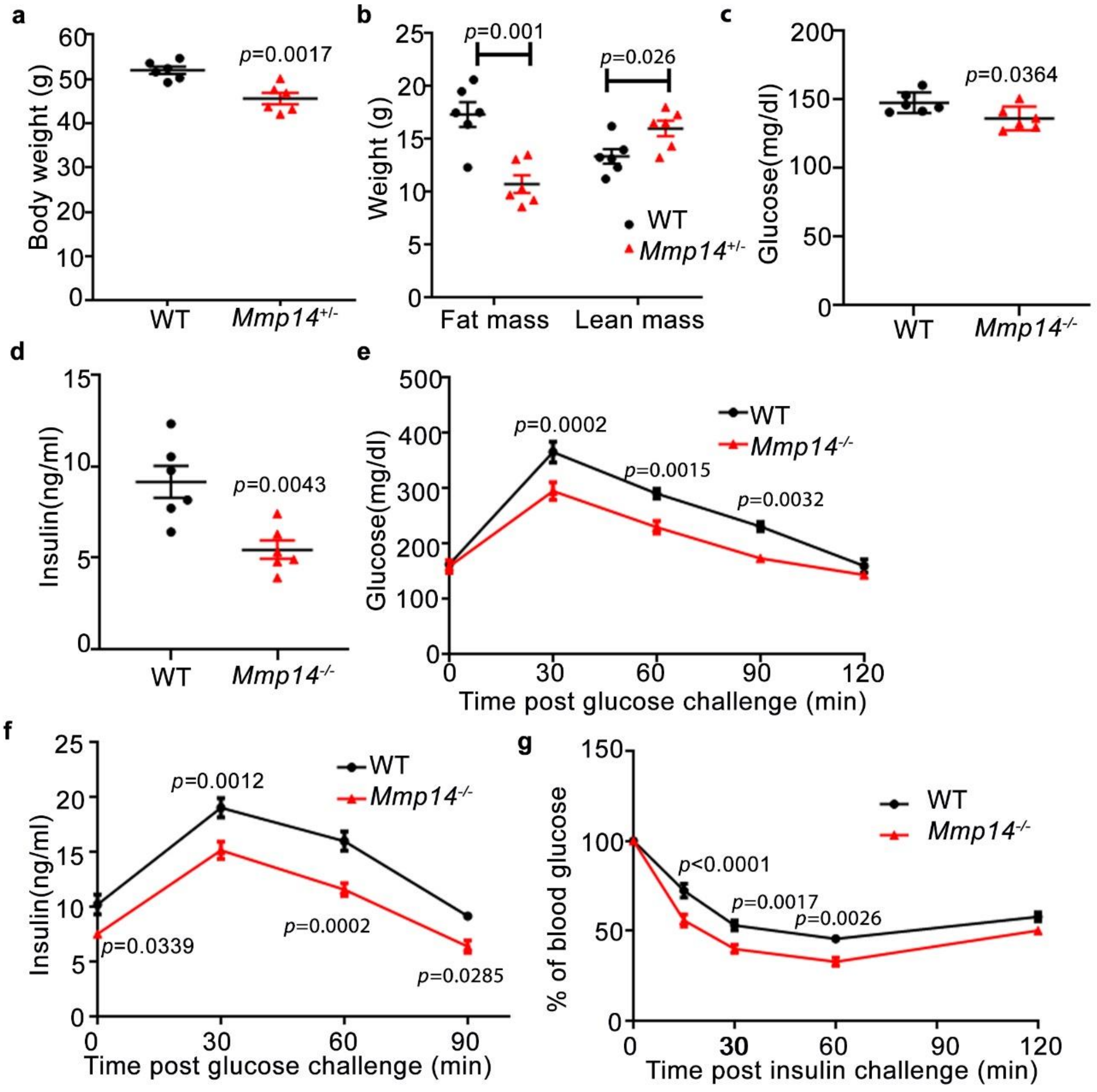

Supplementary Fig. 2. Heterozygous loss of MT1-MMP improves insulin sensitivity in HFD mice

(a-d) Body weight **(a)**, body composition including fat mass and lean mass **(b)**, fasting plasma glucose **(c)** and plasma insulin **(d)** in WT and *Mmp14*$^{+/-}$ mice on high fat diet at the age of 5 months. (n=6) **(e)** Plasma glucose level in WT and *Mmp14*$^{+/-}$ mice on high fat diet during the intraperitoneal glucose tolerance tests. (n=6) **(f)** Plasma insulin level in WT and *Mmp14*$^{+/-}$ mice on high fat diet during the intraperitoneal glucose tolerance tests. (n=6) **(g)** Plasma glucose level in WT and *Mmp14*$^{+/-}$ mice on high fat diet during the insulin tolerance tests. (n=5); In these experiments, the mice were studied at 20 weeks of age. Values are expressed as mean ± SEM; two-tailed unpaired t-test for **(a-d);** two-way ANOVA for **(e-g)**. Source data are provided as a Source Data file.

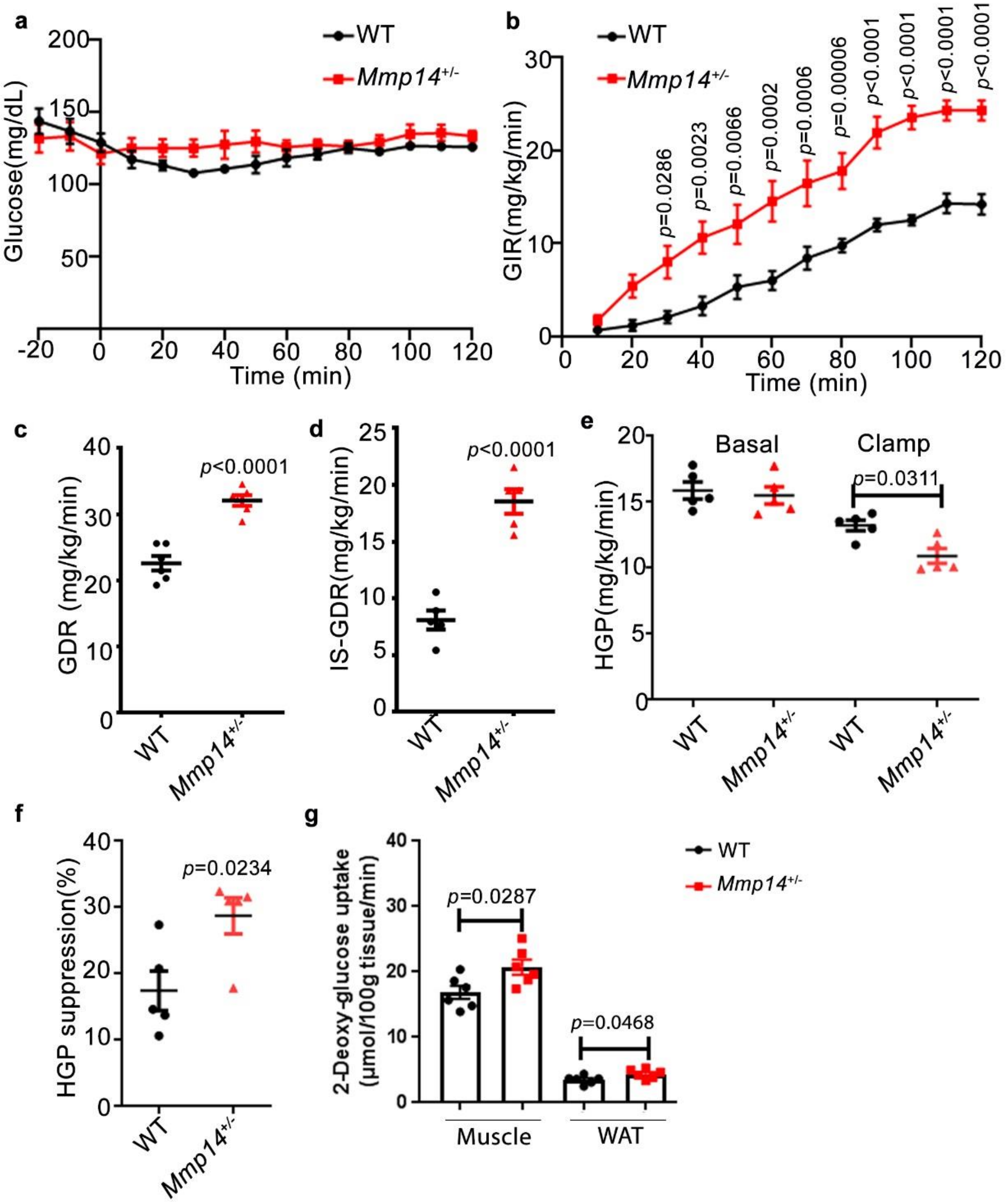

Supplementary Fig. 3. Clamp studies on *Mmp14*$^{+/-}$ mice on high fat diet

(a-g) Plasma glucose **(a)** (n=6), glucose infusion rate (GIR) **(b)** (n=6), glucose disposal rate (GDR) **(c)** (n=6), insulin-stimulated glucose disposal rate (IS-GDR) **(d)** (n=5), hepatic glucose production (HGP) **(e)** (n=5), suppression of HGP during clamp **(f)** (n=5) and glucose uptake in specific tissues **(g)** (n=6) in WT and *Mmp14*$^{+/-}$ mice with high fat diet-induced obesity at the age of 5 month. In these experiments, the mice were studied at 20 weeks of age. Values are expressed as mean ± SEM; two-way ANOVA for **(b &e)**, two-tailed unpaired t-test for **(c-d, f-g).** Source data are provided as a Source Data file.

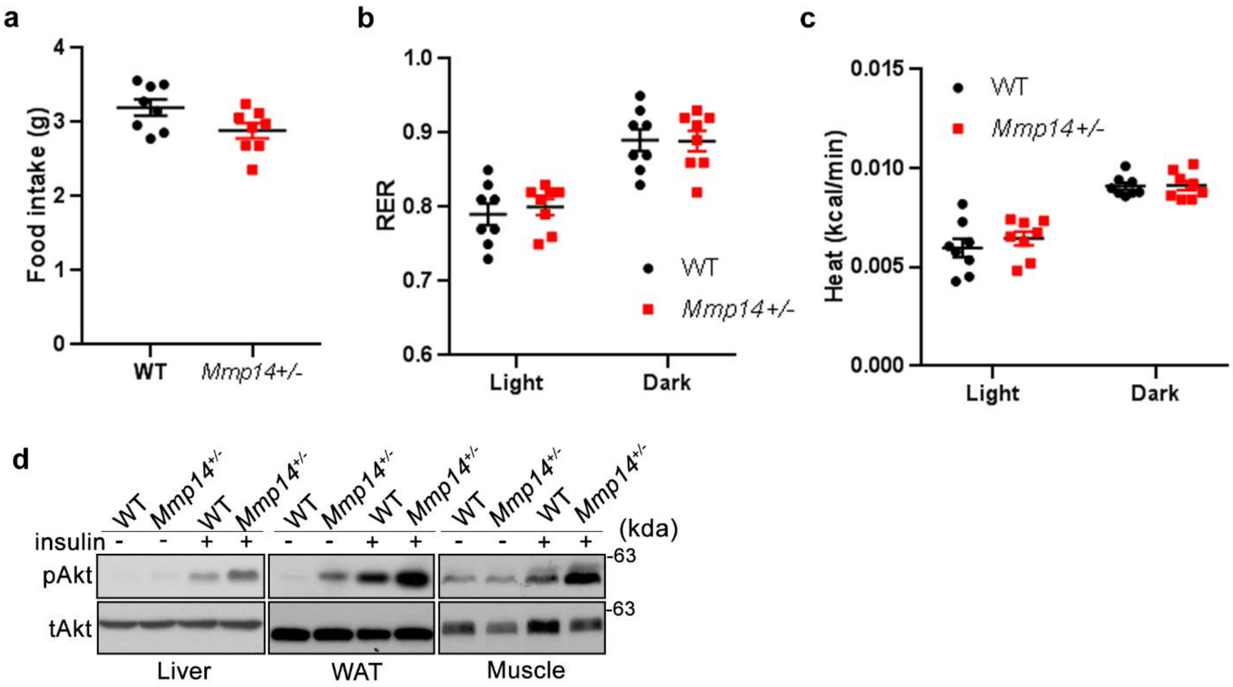

Supplementary Fig. 4. Improved insulin sensitivity in aged *Mmp14*$^{+/-}$ mice

(a) Food intake in wild-type and *Mmp14*$^{+/-}$ mice at 18 months of age (n=8). **(b-c)** Indirect calorimetry studies were performed to measure respiratory exchange ratio (RER) **(b)** and heat production **(c).** (n=8) Representative images show western blotting analyses on the basal and insulin-activated levels of phosphorylated Akt and total Akt in the liver, white adipose tissue and skeletal muscle from wild-type and *Mmp14*$^{+/-}$ mice at 18 months of age after the mice were infused with insulin. (n=3) Quantification of pAkt expression in relative to tAkt expression was shown in **Fig. 1F**. Data are reported as average ± s.e.m. Source data are provided as a Source Data file.

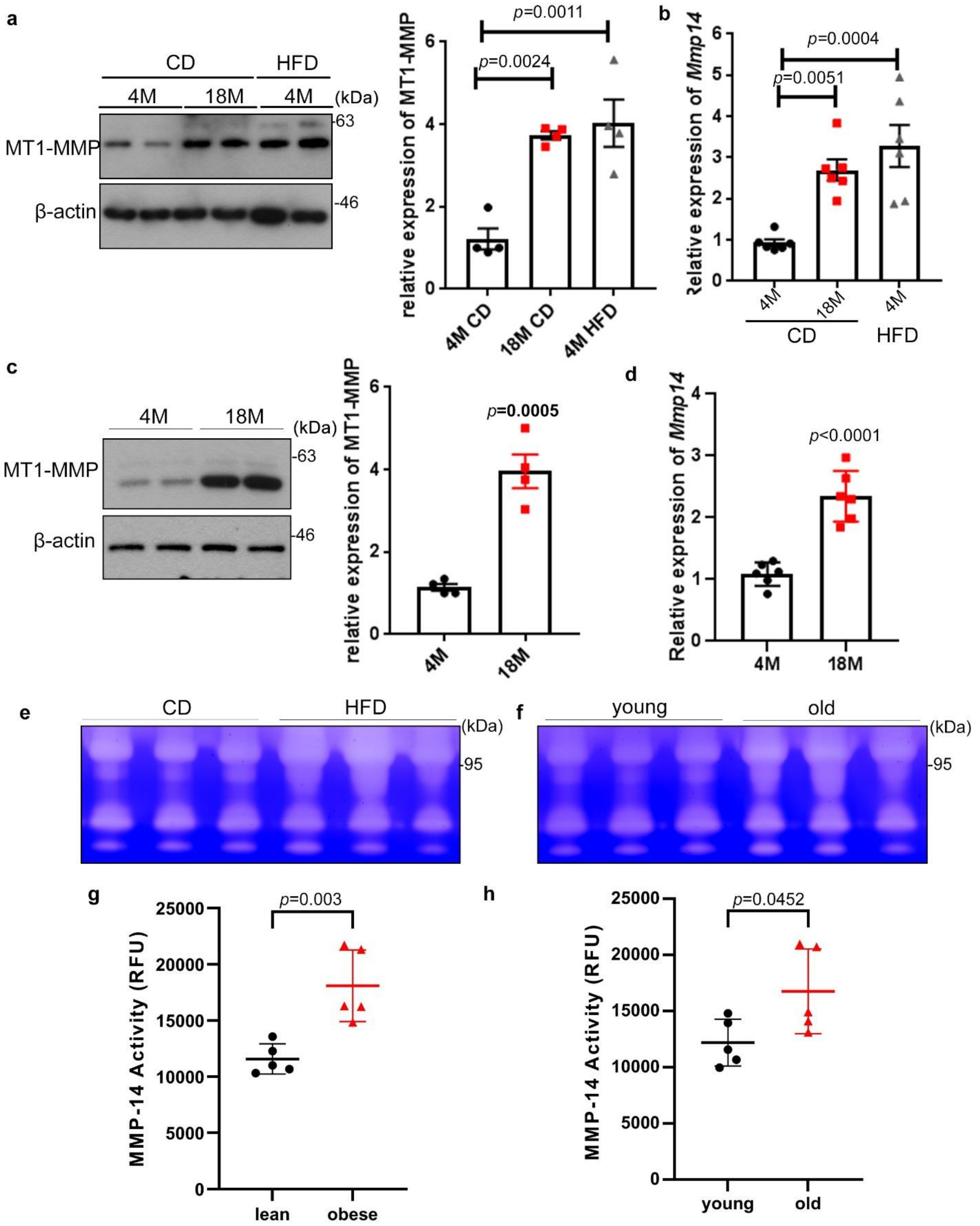

Supplementary Fig. 5. MT1-MMP activation in mice on HFD and aged mice

(a) Western blotting analyses on the expression of MT1-MMP in the livers from high fat diet-fed mice at 4 months of age and from chow-fed mice at 4 months and 18 months of age (left panel). Quantification of relative MT1-MMP expression in relative to β-actin expression was shown in

the right panel (n=4) **(b)** The mRNA expression of MT1-MMP in the livers from high fat diet-fed mice at 4 months of age and from chow-fed mice at 4 months and 18 months of age (n=6). **(c-d)** The expression of MT1-MMP in white adipose tissue (WAT) from chow-fed mice and high fat diet-fed mice at 4 months was examined by western blotting **(c, right panel)** and qPCR analyses **(d)**. Quantification of relative MT1-MMP expression in relative to β-actin expression was shown in **(c, left panel)** (n=4). **(e-f)** Representative images of gel zymography showing the activity of MMP in the liver of mice fed a high fat diet (HFD) and a chow diet (CD) at 4 months of age **(e)** (n=4) and in the liver from chow-fed mice at 4 months (young) and 18 months (old) of age **(f)** (n=4). **(g-h)** The activity of MT1-MMP was measured by using specific fluorescently quenched MMP14 substrates in the liver of mice fed a high fat diet (HFD) and a chow diet at 4 months of age **(g)** and in the liver of mice from chow-fed mice at 4 months (young) and 18 months (old) of age **(h)** (n=5) Values are expressed as mean ± SEM. one-way ANOVA for **(a-b)**, two-tailed unpaired t-test for **(c-d, g-h).** Source data are provided as a Source Data file.

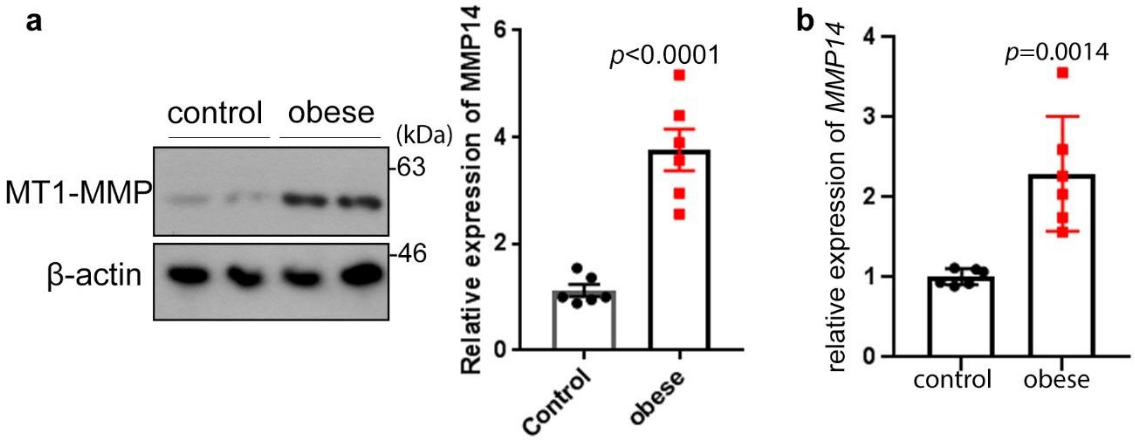

Supplementary Fig. 6. Upregulation of MT1-MMP in obese humans

(a-b) Western blotting **(a, left panel)** and qPCR analyses **(b)** on the expression of MMP14 in the white adipose tissues from lean and obese human subjects with the average age of 29.2 years. Quantification of relative MT1-MMP protein expression in relative to β-actin expression was shown in **(a, right panel)** (n=6 for each group). Values are expressed as mean ± SEM; two-sided unpaired t-test. Source data are provided as a Source Data file.

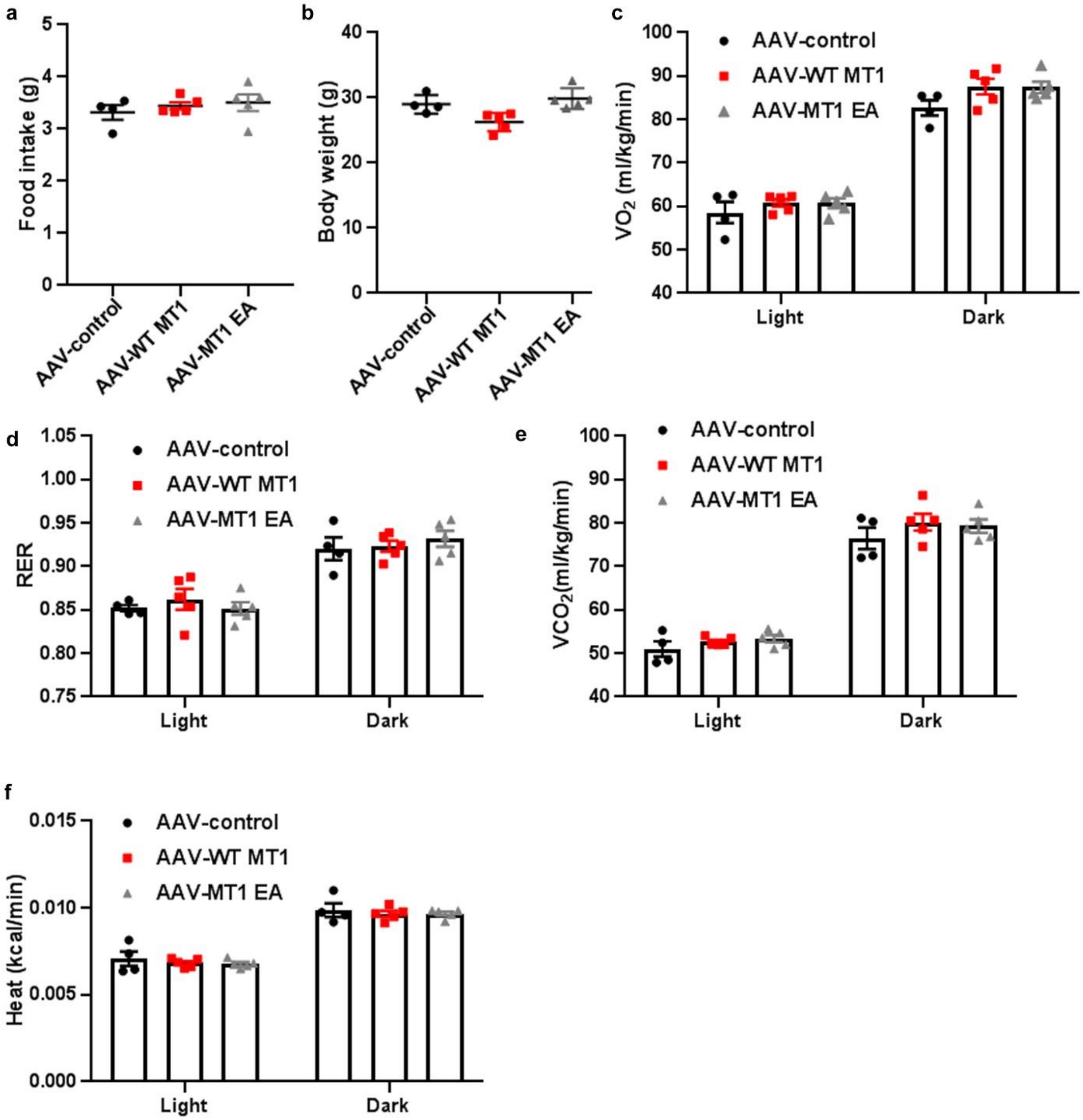

Supplementary Fig. 7. Body parameters in mice receiving AAV-WT MT1
(a-b) Food intake **(a)** and body weight **(b)** in AAV-WT MT1 or AAV-MT1 EA injected mice that were fed standard chow diet at the age of 12 weeks. Indirect calorimetry studies were performed to evaluate VO_2 **(d)**, respiratory exchange ratio (RER) **(d)**, VCO_2 **(e)** and heat **(f)**. (n=4 for AAV-control; n=5 for AAV-WT MT1 & AAV-MT1 EA) Values are expressed as mean ± SEM. Source data are provided as a Source Data file.

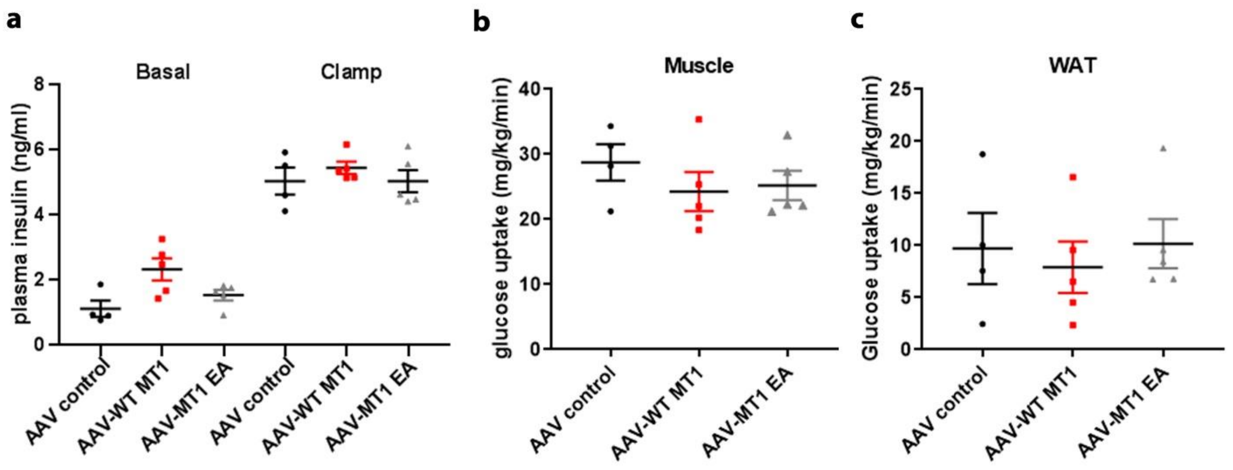

Supplementary Fig. 8. Clamp studies in mice receiving AAV-WT MT1
(a-c) Plasma insulin during the clamp study **(a)** and glucose update in skeletal muscle **(b)** and white adipose tissues (WAT) **(C)** in AV-WT MT1 or AAV-MT1 EA injected mice that were fed standard chow diet at the age of 12 weeks. (n=5 for each treatment group) Values are expressed as mean ± SEM. Source data are provided as a Source Data file.

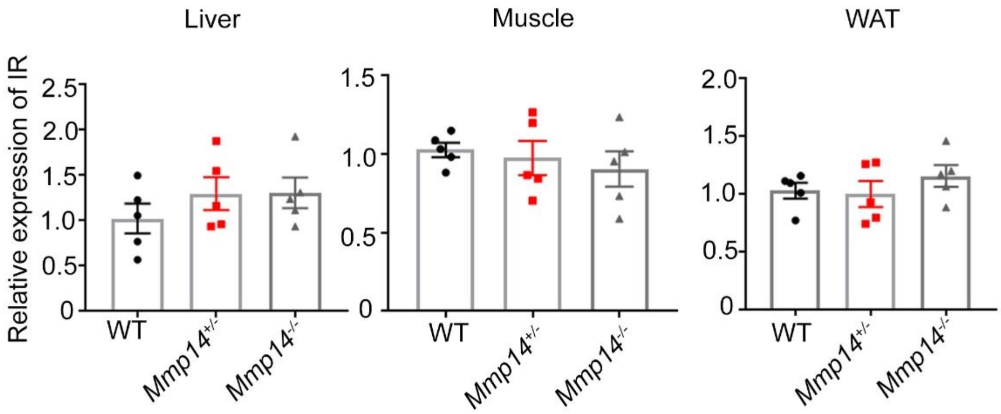

Supplementary Fig. 9. Loss of MT1-MMP does not alter the transcriptional expression of insulin receptor in mice

qPCR analyses on the expression of *Mmp14* in the livers and muscles from wild-type (WT), *Mmp14*$^{+/-}$ and *Mmp14*$^{-/-}$ mice at p15 (n=5) Values are expressed as mean ± SEM. Source data are provided as a Source Data file.

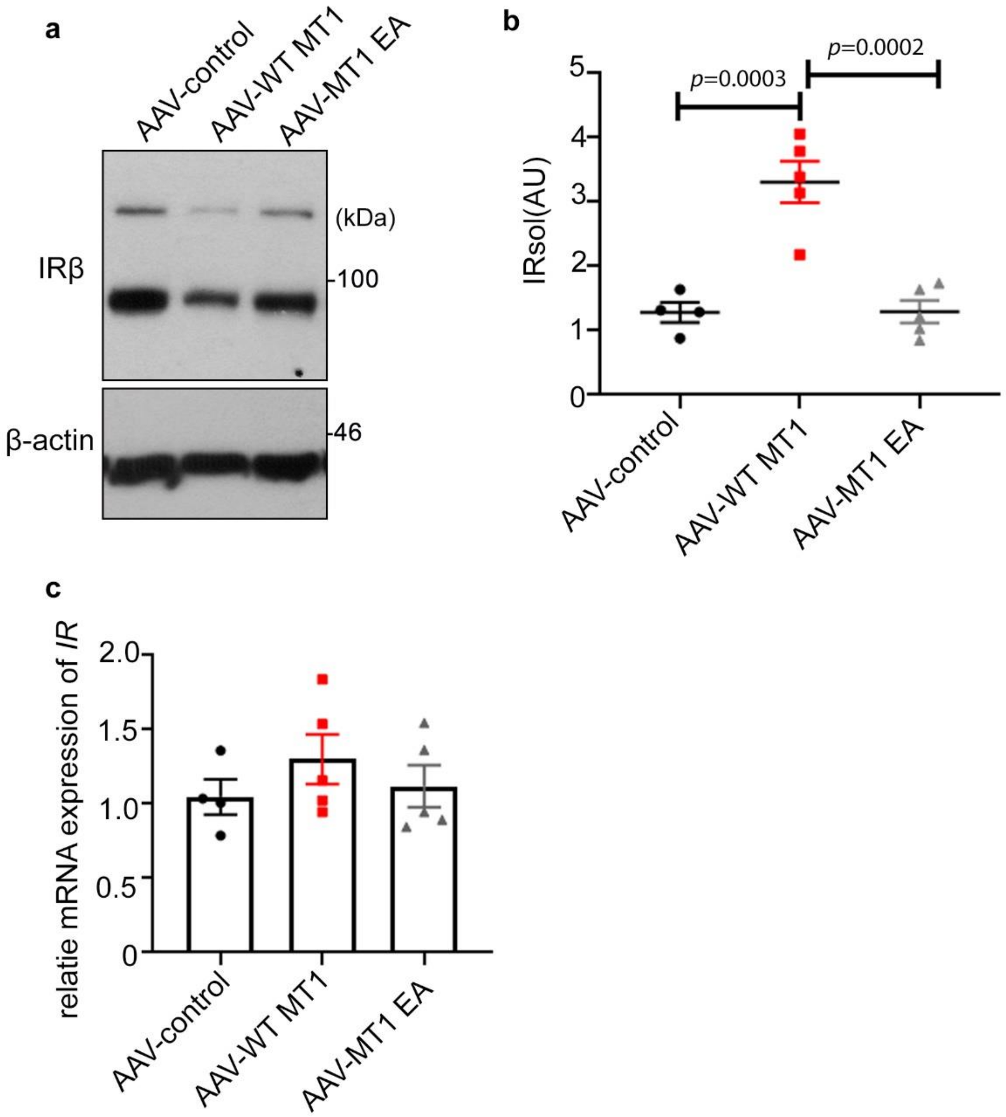

Supplementary Fig. 10 Ectopic expression of MT1-MMP in the liver promotes the release of sIR

12-week-old male mice were injected intravenously with AAV and fed on regular diet **(a)** Western blotting analyses on the expression of IR in the livers from mice receiving either AAV-control, AAV WT MT1 or AAV MT1 EA at the age of 12 weeks. (n=5) **(b)** The level of sIR was measured in the plasma from mice receiving either AAV-control, AAV WT MT1 or AAV MT1 EA. (n=5) **(c)** The relative mRNA expression of IR in the livers from mice receiving either AAV-control, AAV WT MT1 or AAV MT1 EA was analyzed by qPCR. (n=5) Values are expressed as mean ± SEM; two-way ANOVA. Source data are provided as a Source Data file.

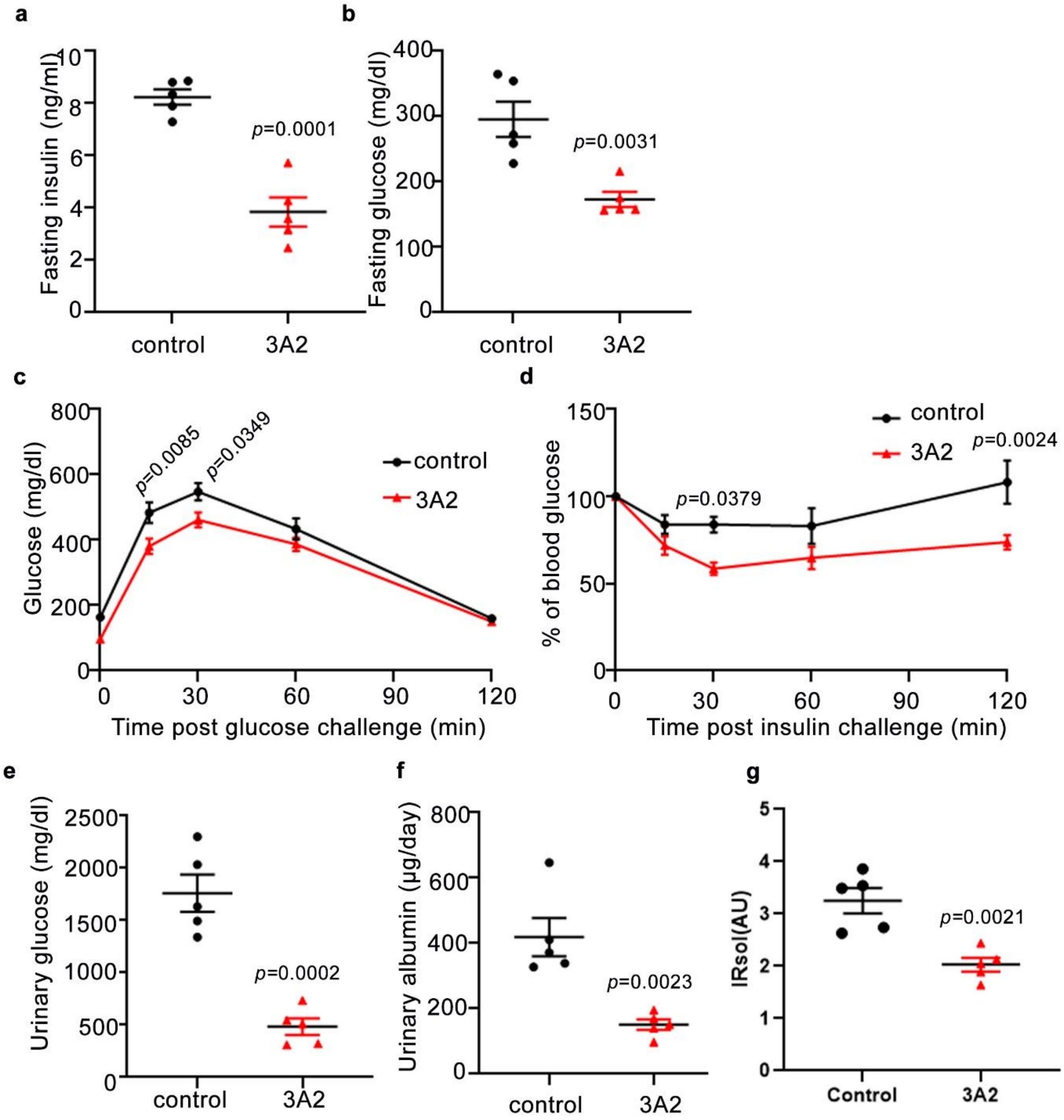

Supplementary Fig. 11 Inhibition of MT1-MMP improves glucose responses in diabetic mice

(a-f) Plasma insulin **(a)** Plasma glucose **(b)** glucose tolerance test **(c)** Insulin tolerance test **(d)** Urinary glucose levels **(e)** Urinary albumin levels **(f)** the level of sIR in the plasma **(g)** in 3 months old db/db male mice receiving treatment (twice a week) with either 3A2 antibody or control IgG for 4 weeks. (n=5 for each group in all experiments) Values are expressed as mean ± SEM; two-way ANOVA for **(c-d)**, two-sided unpaired t-test for **(a-b, e-g)**. Source data are provided as a Source Data file.

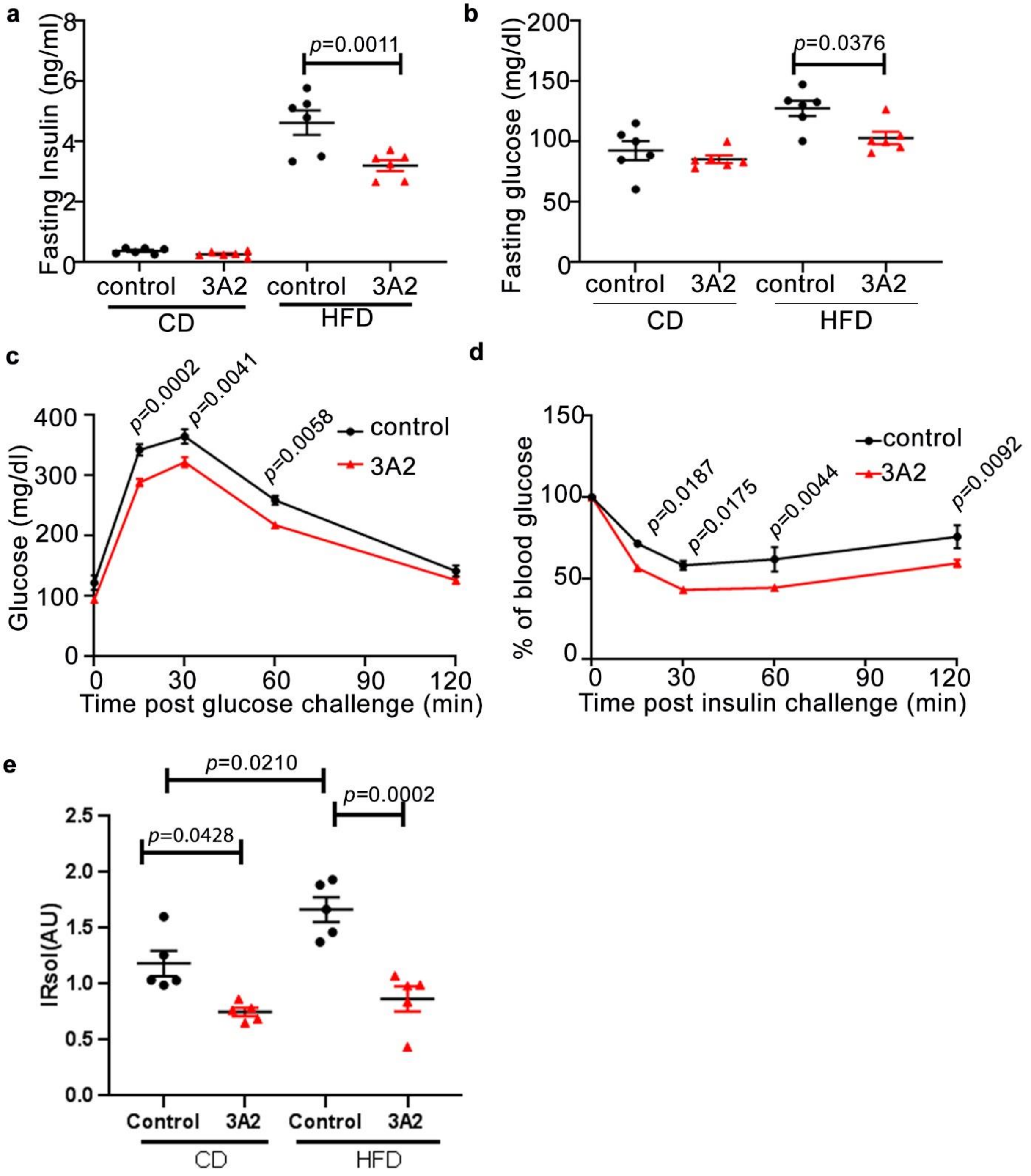

Supplementary Fig. 12 Inhibition of MT1-MMP improves glucose responses in mice with diet induced obesity

8 weeks old male C57BL/6 mice were fed HFD or control chow diet (CD) for 4 weeks and then injected (twice a week) with either 3A2 antibody or control IgG for 4 weeks. **(a-e)** Plasma insulin **(a)** Plasma glucose **(b)** glucose tolerance test **(c)** Insulin tolerance test **(d)** the level of sIR in the plasma **(e)** (n=5 for each group in all experiments) Values are expressed as mean ± SEM; two-way ANOVA. Source data are provided as a Source Data file.

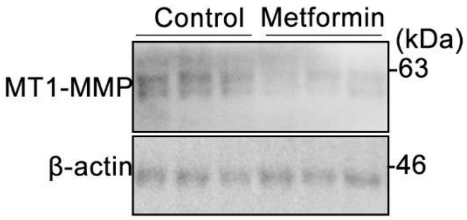

Supplementary Fig. 13 Metformin reduces the expression of MT1-MMP in obese mice
6 months old male C57BL/6 mice with high fat diet-induced obesity were daily treated with metformin (300mg/kg) by oral administration for two weeks. Representative images show western blotting analyses on the expression of MT1-MMP in the liver (n=4). Source data are provided as a Source Data file.
