# Supplementary material for: Regulation of age-associated insulin resistance by MT1-MMP-mediated cleavage of insulin receptor
Source: Nat Commun. 2022 Jun 29;13:3749. doi: 10.1038/s41467-022-31563-2 (PMC9242991; doi:10.1038/s41467-022-31563-2)
Supplement: Supplementary file 2 — Reporting Summary [file 41467_2022_31563_MOESM2_ESM.pdf]

nature portfolio

Corresponding author(s): Wong Hoi Ieong Xavier, Bian Zhaoxiang

Last updated by author(s): May 12, 2022

Reporting Summary

Nature Portfolio wishes to improve the reproducibility of the work that we publish. This form provides structure for consistency and transparency in reporting. For further information on Nature Portfolio policies, see our Editorial Policies and the Editorial Policy Checklist.

Statistics

For all statistical analyses, confirm that the following items are present in the figure legend, table legend, main text, or Methods section.

n/a	Confirmed	
☐	☒	The exact sample size (*n*) for each experimental group/condition, given as a discrete number and unit of measurement
☐	☒	A statement on whether measurements were taken from distinct samples or whether the same sample was measured repeatedly
☐	☒	The statistical test(s) used AND whether they are one- or two-sided<br>*Only common tests should be described solely by name; describe more complex techniques in the Methods section.*
☒	☐	A description of all covariates tested
☐	☒	A description of any assumptions or corrections, such as tests of normality and adjustment for multiple comparisons
☐	☒	A full description of the statistical parameters including central tendency (e.g. means) or other basic estimates (e.g. regression coefficient) AND variation (e.g. standard deviation) or associated estimates of uncertainty (e.g. confidence intervals)
☐	☒	For null hypothesis testing, the test statistic (e.g. *F*, *t*, *r*) with confidence intervals, effect sizes, degrees of freedom and *P* value noted<br>*Give P values as exact values whenever suitable.*
☒	☐	For Bayesian analysis, information on the choice of priors and Markov chain Monte Carlo settings
☒	☐	For hierarchical and complex designs, identification of the appropriate level for tests and full reporting of outcomes
☐	☒	Estimates of effect sizes (e.g. Cohen's *d*, Pearson's *r*), indicating how they were calculated

Our web collection on statistics for biologists contains articles on many of the points above.

Software and code

Policy information about availability of computer code

Data collection	qPCR analyses were performed by the ABI ViiA 7 real-time PCR system (Applied Biosystems).
Data analysis	GraphPad Prism V8 for Window OS was used for statistical analyses.<br>Protein bands of western blots were quantified using Image J (version 1.8.0_172).<br>qPCR results were analyzed by ViiA 7 Real-time PCR system software (QuantStudio Software v1.6.1)

For manuscripts utilizing custom algorithms or software that are central to the research but not yet described in published literature, software must be made available to editors and reviewers. We strongly encourage code deposition in a community repository (e.g. GitHub). See the Nature Portfolio guidelines for submitting code & software for further information.

Data

Policy information about availability of data

All manuscripts must include a data availability statement. This statement should provide the following information, where applicable:

- Accession codes, unique identifiers, or web links for publicly available datasets
- A description of any restrictions on data availability
- For clinical datasets or third party data, please ensure that the statement adheres to our policy

All data generated or analysed during this study are included in this published article (and its supplementary information files).

Field-specific reporting

Please select the one below that is the best fit for your research. If you are not sure, read the appropriate sections before making your selection.

- [x] Life sciences
- [ ] Behavioural & social sciences
- [ ] Ecological, evolutionary & environmental sciences

For a reference copy of the document with all sections, see nature.com/documents/nr-reporting-summary-flat.pdf

Life sciences study design

All studies must disclose on these points even when the disclosure is negative.

Sample size	We selected the sample size, based on our extensive experience with animal models and endpoints. We make sure that no more animals than necessary were used. Sample size were chosen to generate reproducible results with desirable significance (0.05) and power (>90%).<br>For in vitro experiments, we did not compute statistical analyses to predetermine sample sizes. The sample sizes (at lease three independent experimental replicates) were chosen based on the standard practice of the research field.
Data exclusions	No data was excluded from the manuscript.
Replication	All of the experimental results were replicated as indicated in figure legends. For in vitro experiments, each experiment was independently repeated at least three times. Only biological replicates were plotted and used for statistical analyses.
Randomization	For in vivo experiments, tissues from independently and randomly chosen mice at comparable developmental stages and sexes were collected for analyses and none of the samples was excluded. For the experiments involving transgenic animals, further allocations were based on the genotype of mice. For in vitro experiments, groups were allocated based on the genetic background of cells and different treatments. Therefore, no randomization was required.
Blinding	During the phenotyping experiments, such as body weight and food intake measurement, glucose tolerance test and insulin sensitivity testing, the experiments were performed blinded and the genotype was only disclosed after data analyses.<br>For molecular studies including western blotting and qPCR analyses, the investigators were not blinded to experimental conditions for planning experiments, due to careful experimental setup and the complexity of the experiments. The samples had to be loaded in an appropriate order for better data comparison.

Reporting for specific materials, systems and methods

We require information from authors about some types of materials, experimental systems and methods used in many studies. Here, indicate whether each material, system or method listed is relevant to your study. If you are not sure if a list item applies to your research, read the appropriate section before selecting a response.

Materials & experimental systems

n/a	Involved in the study	
[ ]	[x]	Antibodies
[ ]	[x]	Eukaryotic cell lines
[x]	[ ]	Palaeontology and archaeology
[ ]	[x]	Animals and other organisms
[ ]	[x]	Human research participants
[x]	[ ]	Clinical data
[x]	[ ]	Dual use research of concern

Methods

n/a	Involved in the study	
[x]	[ ]	ChIP-seq
[x]	[ ]	Flow cytometry
[x]	[ ]	MRI-based neuroimaging

Antibodies

Antibodies used	anti-MT1-MMP antibody (ab51074, Abcam; 1:2000 for western blotting); anti-insulin Rα antibody (sc-57344, Santa Cruz, 1:2000 for western blotting); anti-insulin Rβ antibody (sc-711, Santa Cruz, 1:2000 for western blotting); anti-insulin Rβ antibody (clone CT-3, MAB S65, millipore, 1:2000 for western blotting) anti-Akt (4685, Cell Signaling, 1:3000 for western blotting): anti-pAkt (4060, Cell Signaling, 1:2000 for western blotting); anti-β-actin (12262, Cell Signaling, 1:5000 for western blotting); goat anti-rabbit antibody conjugated with HRP (sc-2030, Santa Cruz, 1:2000); Rabbit anti-mouse antibody conjugated with HRP (sc-358914, Santa Cruz, 1:2000)
Validation	anti-MT1-MMP antibody (ab51074, Abcam), validated for IHC-P, IP, Flow cytometry, WB, IF<br>https://www.abcam.com/mmp14-antibody-ep1264y-ab51074.html<br><br>anti-insulin Rα antibody (sc-57344, Santa Cruz), validated for IP, WB<br>https://www.scbt.com/p/insulin-ralpha-antibody-ma-20<br><br>anti-insulin Rβ antibody (sc-711, Santa Cruz, discontinued), validated for IP, WB

https://www.scbt.com/p/insulin-rbeta-antibody-c-19

anti-insulin Rβ antibody (clone CT-3, MAB S65, Sigma, 1:2000 for western blotting), validated for IP, WB, IHC
https://www.merckmillipore.com/HK/en/product/Anti-Insulin-Receptor-Antibody-beta-subunit-clone-CT-3,MM_NF-MABS65?ReferrerURL=https%3A%2F%2Fwww.google.com%2F&bd=1

anti-Akt (4685, Cell Signaling), validated by the manufacturer for WB, IF, IHC, Flow
https://www.cellsignal.com/products/primary-antibodies/akt-pan-11e7-rabbit-mab/4685?_=1641294519526&Ntt=4685&tahead=true

anti-pAkt (Ser473) (4060, Cell Signaling), validated by the manufacturer for WB, IP. IHC, IF, Flow
https://www.cellsignal.com/products/primary-antibodies/phospho-akt-ser473-d9e-xp-rabbit-mab/4060

anti-β-actin (HRP conjugate) (12262, Cell Signaling), validated by the manufacturer for WB
https://www.cellsignal.com/products/antibody-conjugates/b-actin-8h10d10-mouse-mab-hrp-conjugate/12262?_=1641294860966&Ntt=12262&tahead=true,

goat anti-rabbit antibody conjugated with HRP (sc-2030, Santa Cruz), validated by the manufacturer for WB.
https://www.scbt.com/p/goat-anti-rabbit-igg-hrp-cruz-marker-compatible

Rabbit anti-mouse antibody conjugated with HRP (sc-358914, Santa Cruz), validated by the manufacturer for WB.
https://www.scbt.com/p/rabbit-anti-mouse-igg-hrp

Eukaryotic cell lines

Policy information about cell lines

Cell line source(s)	HEK293T cells were provided by Professor Zhou Zhongjun at the University of Hong Kong.<br>HEK293T cells are commercially available in ATCC<br>https://www.atcc.org/products/crl-3216
Authentication	Cell lines were not authenticated.
Mycoplasma contamination	The cells were tested negative for mycoplasma contamination
Commonly misidentified lines<br>(See ICLAC register)	No commonly misidentified cell lines were used

Animals and other organisms

Policy information about studies involving animals; ARRIVE guidelines recommended for reporting animal research

Laboratory animals	Wild-type mice on C57BL/6J background were from the Laboratory Animal Services Centre of The Chinese University of Hong Kong. Mmp14+/- mice on C57BL/6J background were a kind gift from Prof. Zhou Zhongjun in the University of Hong Kong. All animal experiments involves animals with starting age of 4 weeks. As MT1-MMP deficient mice die prematurely 20 days after birth, the animal experiments involving this mouse model would make use of mice at p15. Animals of both sexes were used in the experiments unless it was specifically stated in the figure ligand. All animals and their borne pups were housed in the animal house at Hong Kong Baptist University and maintained on a 12-hour (h) light/dark cycle with constant ambient temperature (22°C – 24°C) and humidity (~60%).<br><br>For the non-human primate ageing model, young (mean=3.8 years) and old (mean=18.4 years) cynomolgus macaques were used. They were all female and healthy.
Wild animals	No wild animals was used.
Field-collected samples	This study did not involve samples collected from the fields.
Ethics oversight	All mouse experiments were approved by the Committee on the Use of Human & Animal Subjects in Teaching & Research (HASC) at Hong Kong Baptist University and procedures were approved by the Department of Health under Hong Kong legislation.<br><br>For the monkey study, all housing conditions and procedures were approved by and in compliance with the ethical guideline of the Institutional Animal Care and Use Committee (IACUC) of Guangzhou Huazhen Biosciences Co., Ltd in Guang Zhou, Gunag dong, China. The housing facilities was accredited by the Association for Assessment and Accreditation of Laboratory Animal Care (AAALAC).

Note that full information on the approval of the study protocol must also be provided in the manuscript.

Human research participants

Policy information about studies involving human research participants

Population characteristics	Young (mean=29.2 years) and elderly (mean=80.1 years) human subjects (men or women), mainly of Asian Chinese, were recruited from Guanfu Hospital in Jinhua, Zhejiang Province, China. They were healthy and non-obese with BMI<24.
Recruitment	Human subjects, mainly of Chinese ethnicity, were recruited from Guanfu Hospital in Jinhua, Zhejiang Province, China. Potential subjects were selected from the outpatient pool with normal BMI index. After signing the screening consent, human subjects were asked to fill in a screening questionnaire to assess their heath and medication history, including physical activity. Those subjects who had no long-term illness and met the inclusion criteria and were willing to participate in the study were invited for blood collection for further analyses.
Ethics oversight	The study protocol was approved by the Research Ethics Board of Guanfu Hospital and the Health Commission of Guangdong Province in accordance with China legislation. The study was conducted in accordance with the Declaration of Helsinki.

Note that full information on the approval of the study protocol must also be provided in the manuscript.
